# CD161 expression and regulation defines rapidly responding effector CD4+ T cells associated with improved survival in HPV16-associated tumors

**DOI:** 10.1136/jitc-2021-003995

**Published:** 2022-01-17

**Authors:** Chantal L Duurland, Saskia J Santegoets, Ziena Abdulrahman, Nikki M Loof, Gregor Sturm, Tom H Wesselink, Ramon Arens, Sanne Boekestijn, Ilina Ehsan, Mariette I E van Poelgeest, Francesca Finotello, Hubert Hackl, Zlatko Trajanoski, Peter ten Dijke, Veronique M Braud, Marij J P Welters, Sjoerd H van der Burg

**Affiliations:** 1 Department of Medical Oncology, Oncode Institute, Leiden University Medical Center, Leiden, The Netherlands; 2 Biocenter, Institute of Bioinformatics, Medical University of Innsbruck, Innsbruck, Austria; 3 Department of Immunology, Leiden University Medical Center, Leiden, The Netherlands; 4 Department of Gynecology, Leiden University Medical Center, Leiden, The Netherlands; 5 Institute of Molecular Biology, University of Innsbruck, Innsbruck, Austria; 6 Digital Science Center (DiSC), University of Innsbruck, Innsbruck, Austria; 7 Department of Cell and Chemical Biology, Leiden University Medical Center, Leiden, The Netherlands; 8 Institut de Pharmacologie Moléculaire et Cellulaire, Centre National de la Recherche Scientifique, Université Côte d’Azur, UMR7275, 06560 Valbonne, Sophia Antipolis, France

**Keywords:** T-lymphocytes, lymphocyte activation, lymphocytes, tumor-infiltrating, tumor microenvironment, immunity, cellular

## Abstract

**Background:**

Expression of killer cell lectin-like receptor B1 (*KLRB1*), the gene encoding the cell surface molecule CD161, is associated with favorable prognosis in many cancers. CD161 is expressed by several lymphocyte populations, but its role and regulation on tumor-specific CD4+ T cells is unknown.

**Methods:**

We examined the clinical impact of CD4+CD161+ T cells in human papillomavirus (HPV)16+ oropharyngeal squamous cell carcinoma (OPSCC), analyzed their contribution in a cohort of therapeutically vaccinated patients and used HPV16-specific CD4+CD161+ tumor-infiltrating lymphocytes and T cell clones for in-depth mechanistic studies.

**Results:**

Central and effector memory CD4+ T cells express CD161, but only CD4+CD161+ effector memory T cells (Tem) are associated with improved survival in OPSCC. Therapeutic vaccination activates and expands type 1 cytokine-producing CD4+CD161+ effector T cells. The expression of CD161 is dynamic and follows a pattern opposite of the checkpoint molecules PD1 and CD39. CD161 did not function as an immune checkpoint molecule as demonstrated using multiple experimental approaches using antibodies to block CD161 and gene editing to knockout CD161 expression. Single-cell transcriptomics revealed *KLRB1* expression in many T cell clusters suggesting differences in their activation. Indeed, CD4+CD161+ effector cells specifically expressed the transcriptional transactivator *SOX4,* known to enhance T cell receptor (TCR) signaling via CD3ε. Consistent with this observation, CD4+CD161+ cells respond more vigorously to limiting amounts of cognate antigen in presence of interleukin (IL)-12 and IL-18 compared to their CD161- counterparts. The expression of CD161/*KLRB1* and *SOX4* was downregulated upon TCR stimulation and this effect was boosted by transforming growth factor (TGF)β1.

**Conclusion:**

High levels of CD4+CD161+ Tem are associated with improved survival and our data show that CD161 is dynamically regulated by cell intrinsic and extrinsic factors. CD161 expressing CD4+ T cells rapidly respond to suboptimal antigen stimulation suggesting that CD161, similar to SOX4, is involved in the amplification of TCR signals in CD4+ T cells.

## Background

Infection with human papillomavirus type 16 (HPV16) can cause tumors in the oropharynx and cervix.^1^ Presence of an intratumoral type 1 T cell response against the viral oncoproteins E6 and E7 (immune response (IR)+) is strongly associated with improved survival in oropharyngeal squamous cell carcinoma (OPSCC). Mass cytometry analysis of the tumor microenvironment (TME) revealed that HPV16+IR+ OPSCC and cervical cancer were highly infiltrated by CD4+CD161+ effector cells.^2 3^

CD161 is a C-type lectin receptor expressed on natural killer (NK) cells and T cells in peripheral blood, umbilical cord blood, and thymus.^4 5^ (Tumor-specific) CD4+CD161+ and CD8+CD161+ cells produce more pro-inflammatory cytokines compared to CD161- cells.^2 3 6 7^ Transcriptional profling of CD4+, CD8+ and γδ+ T cells expressing CD161 identified a shared transcriptional profile and innate-like function among these cell lineages.^8^ Lectin-like transcript 1 (LLT1), the ligand for CD161, is expressed by activated B cells and dendritic cells (DC), but also malignant cells.^9–14^ CD161 functions as a coinhibitory receptor on NK cells, but on T cells the function is less clear as studies reported a costimulatory, coinhibitory, or no function for CD161.^8 10 12 13 15–17^

In this study, we examined the role and regulation of CD161 expression on tumor-specific CD4+ cells. Our data show that CD4+CD161+ effector memory T cells (Tem) are associated with improved survival and can be activated upon therapeutic vaccination. CD161 does not function as an immune checkpoint molecule on CD4+ cells. Instead, CD161 expression on CD4+ cells is dynamically regulated and coexpressed with the transcriptional transactivator *SOX4,* and CD4+CD161+ cells respond more vigorously to cognate antigen stimulation under suboptimal conditions.

## Materials and methods

### Patient samples

All patients received standard-of-care treatment and HPV typing was performed.^18^ Formalin-fixed paraffin-embedded (FFPE) biopsies of 40 histologically confirmed OPSCC patients were included (HPV16+IR+ n=25, HPV16+IR- n=15). Peripheral blood mononuclear cells (PBMC) were isolated from venous blood of healthy donors and patients using ficoll density gradient centrifugation, cryopreserved and stored until use.

### Multispectral immunofluorescence

A multispectral immunofluorescence panel containing CD3, CD8, PD1, CD45RO, CD161 and DAPI (online supplemental table 1) was designed and optimized,^19^ see online supplemental material.

10.1136/jitc-2021-003995.supp1 Supplementary data

10.1136/jitc-2021-003995.supp2 Supplementary data

### Analysis of HPV16-specific T cells after vaccination

PBMC and skin biopsies were collected during studies examining the effect of vaccination in HPV16+ vulvar or vaginal intraepithelial neoplasia.^20 21^ PBMC were obtained before (prevaccination) and 2 weeks postvaccination. Skin biopsies from the vaccination site were obtained 2 weeks postvaccination. Infiltrating T cells were isolated^2^ and cultured in 10% T cell Growth Factor (TCGF, ZeptoMetrix), recombinant human interleukin-7 (rhIL-7), rhIL-15 (5 ng/mL) (PeproTech) and Gentamicin CF (20 µg/mL, Centrafarm). Cells were cryopreserved upon sufficient expansion.

Prevaccination and postvaccination PBMC were prestimulated for 11 days with a pool of 22-mer E6 and E7 (E6E7) peptides (final concentration of each peptide: 2.5 µg/mL) to expand antigen-specific T cells. 10% TCGF and 5 ng/mL rhIL-15 were added after 1 day of culture. Expanded PBMC and cultured T cells were stimulated with monocytes loaded overnight with a pool of 22-mer E6 and E7 peptides (final concentration of each peptide: 5 µg/mL). Monocytes were isolated from autologous PBMC by adherence and cultured with granulocyte-macrophage colony-stimulating factor (GM-CSF) (800 U/mL, Thermo Fisher Scientific (TFS)) for 3 days. Brefeldin A (10 µg/mL, Sigma) was added after 1 hour and cells were incubated overnight before analysis of cytokine production by flow cytometry.

### Flow cytometry and cell sorting

Cells were stained with LIVE-DEAD fixable Yellow or near-IR dead cell stain kit (TFS), incubated with phosphate-buffered saline (PBS) containing 0.5% bovine serum albumin (Sigma) (FACS buffer) and 10% FCS for 10 min at 4°C, washed and stained with fluorochrome-conjugated antibodies (online supplemental table 2) for 20 min at 4°C. Intracellular cytokine production in response to vaccination and rhIL-12 + rhIL-18 was analyzed.^2 3^ Alternatively, cells were fixated and stained intracellularly using BD Cytofix/Cytoperm kit according to manufacturer’s instructions. For LLT1 staining, cells were blocked with 10% goat serum (Dako/Agilent) and 100 µg/mL IgG from human serum (Sigma) in FACS buffer for 30 min, washed, incubated with 10 µg/mL purified mouse-anti-human IgG1 (clone MOPC-21, Biolegend) or LLT1 (clone 4F68, V.M. Braud) for 30 min, washed and incubated with goat-anti-mouse PE (Biolegend) for 30 min. Flow cytometry data were acquired using a BD LSR Fortessa and analyzed using FlowJo software V.10.7.1 (BD).

For cell sorting, cells were used directly after culture, or thawed and enriched for CD4+ cells using the human CD4+ T cell isolation kit (Miltenyi Biotech). Cells were stained with LIVE/DEAD near-IR dead cell stain kit and fluorochrome-conjugated antibodies (online supplemental table 2) as described above, but cells were washed in FACS buffer supplemented with 2 mM EDTA (Sigma). Cells were sorted using BD FACS Aria I or III.

### Killer cell lectin-like receptor B1 (*KLRB1*) gene editing of primary human T cells

Expanded CD4+CD161+ cells from OPSCC tumor-infiltrating lymphocytes (TIL) or HPV16-specific CD4+CD161+ T cell clones (see online supplemental material) were thawed and rested overnight in 10% Iscove’s Modified Dulbecco’s Medium (IMDM) containing rhIL-2 (1000 U/mL, Aldesleukin, Novartis) and rhIL-7 (5 ng/mL) (OPSCC TIL), or rhIL2, rhIL-7 and rhIL-15 (5 ng/mL) (HPV16-specific T cell clones) followed by electroporation with ribonucleoproteins (RNPs) containing guide RNA (gRNA) targeting *KLRB1* or control.^22^ CrisprRNA targeting human *KLRB1*^10^ or control (catalog# 1072544) (Integrated DNA Technologies (IDT)) were suspended to 100 µM in nuclease-free duplex buffer. gRNA were generated by incubating 10 µl of 100 µM crRNA targeting *KLRB1* region#1, *KLRB1* region #2, or control with 10 µL tracrRNA. To prepare the RNP complex, 3 µL annealed gRNA targeting *KLRB1* region#1, *KLRB1* region #2, or control was mixed with 2 µL Cas9 per sample and incubated for 10 min at room temperature. Before nucleofection, cells were washed in PBS and resuspended in 20 µl P2 Nucleofection buffer (Lonza), 1 µL of 100 µM Alt-R cas9 electroporation enhancer (IDT) per nucleofection, and 5 µL control RNP or 2.5 µL KLRB1 region#1 RNP and 2.5 µL KLRB1 region#2 RNP. Mixes were transferred to a 16-well nucleocuvette strip and samples were electroporated using a 4D-nucleofector machine (Lonza) with program EH100. Samples were cultured for 7 days in 10% IMDM containing rhIL-2, rhIL-7 and rhIL-15 as above.

### Functional analysis of CD161

Cells were stimulated with plate-bound anti-CD3 (R&D, clone UCHL1, various concentrations), anti-IgG1 (clone MOPC-21) or anti-CD161 (clone HP-3G10) (Biolegend) (HPV16-specific CD4+ T cell clones: 5 µg/mL, OPSCC TIL: 10 µg/mL). Alternatively, cells were preincubated with biotinylated anti-IgG1 (clone MOPC-21) or anti-CD161 (clone HP-3G10) (Biolegend, 5 µg/mL) for 30 min, washed, and crosslinked with anti-biotin antibody (Stemcell Technologies, 5 µg/mL) during stimulation with plate-bound anti-CD3 (1 µg/mL) and soluble anti-CD28 (2 µg/mL, BD, clone CD28.2). 2 days before coculture, autologous B lymphoblastoid cell lines (B-LCL) were cultured (0.25×10^6^ cells/mL) and after 1 day loaded overnight with indicated amounts of 22-mer E6 and E7 peptides (pool or specific peptide). T cells were preincubated with plate-bound anti-IgG1 or anti-CD161 (10 µg/mL) for 1 hour before coculture with irradiated (7500 RAD) peptide-loaded B-LCL at indicated ratio’s. Cells were cultured for 4 hours in presence of Brefeldin A (10 µg/mL) or 24 hours with Brefeldin A added after 1 hour of culture and analyzed by intracellular cytokine staining, or 3 days and analyzed for interferon γ (IFNγ) and GM-CSF secretion using ELISA (Mabtech).

### T cell stimulation and culture with cycloheximide, transforming growth factor (TGF)β1, Activin A, bone morphogenetic protein (BMP)2, BMP6, rhIL-12, and rhIL-18

CD4+, or sorted CD4+CD8-(CD45RO+)CD161+ or CD161- cells from healthy donors, or HPV16-specific CD4+ T cell clones were cultured directly or first labeled with CellTrace Violet (CTV, 2 µM, TFS). (CTV-labeled) cells were stimulated with anti-CD3CD28 beads (TFS) at indicated bead:cell ratio’s or irradiated (7500 RAD) peptide-loaded B-LCL (B-LCL:T cell is 1:5) for 5 days. Additionally, cells were stimulated with transforming growth factor β1 (TGFβ1) (0, 1, 5 ng/mL, PeproTech) for 5 days or 3 weeks in 10% IMDM and rhIL-7 (healthy donor) or rhIL-15 (clones) (5 ng/mL). TGFβ1 and rhIL-7 were added every 3–4 days to 3-week cultures and cells were restimulated with anti-CD3CD28 every week. For restimulation, cells were harvested and anti-CD3CD28 beads were removed using a magnet before restimulation. TGFβ1, rhIL-7 and rhIL-15 were added to 5-day cultures every 2 days. Cells were preincubated with 1 µM SB505124 (Tocris) or dimethyl sulfoxide (DMSO) for 2 hours followed by 2 hours preincubation with TGFβ1 before adding anti-CD3CD28 beads. Alternatively, TGFβ1 was preincubated with 10 µg/mL pan-neutralizing TGFβ1 (clone 1D11) or control (clone 13C4) antibody for 2 hours in 10% IMDM before preincubation with cells for 2 hours. SB505124, DMSO, pan-neutralizing TGFβ antibody or control, and TGFβ1 were added to cultures every 2 days.

Healthy donor-derived CD4+ cells were cultured with 20 µg/mL cycloheximide (CHX) or DMSO, Activin A ± 1 µM SB505124, bone morphogenetic protein 2 (BMP2) or BMP6 (0, 10, 50, 100, 200 ng/mL) as described for TGFβ1.

HPV16-specific CD4+ T cell clones were stimulated with peptide-loaded B-LCL in presence or absence of 50 ng/mL rhIL-12 (Milteny Biotech) and rhIL-18 (R&D systems). Cells were cultured for 24 hours with 5 µg/mL Brefeldin A during the last 4 hours of culture.

### Imaging mass cytometry (hyperion)

Imaging mass cytometry was performed using an optimized 33-marker panel.^23^ The generated high-dimensional output was analyzed by an in-house developed imaging processing pipeline combining multiple previously validated publicly available software programs (Abdulrahman *et al* submitted), see online supplemental material.

### RNA isolation, qPCR and T cell Receptor analysis

RNA was extracted using Arcturus Picopure RNA isolation kit (TFS) according to manufacturer’s instructions using the protocol for isolation of RNA from cell pellets. cDNA was synthesized using high-capacity RNA-to-cDNA kit (TFS) and amplified using iQ SYBR Green Mastermix (Bio-Rad) and primers (Sigma) against target genes (online supplemental table 3). Relative expression of *KLRB1* upon T cell receptor (TCR) stimulation was normalized to average expression of *BACT* and *B2M*, relative expression of *KLRB1* upon TGFβ1 exposure to *SDHA,* and relative expression of *BACH2*, *EOMES*, *SATB1*, *LEF1*, *TCF7* and *SOX4* to average expression of *B2M* and *SDHA* using the 2^-ΔCq^ method. Expression relative to 0 ng/mL TGFβ1 was calculated using the 2^-ΔΔCq^ method. TCR sequencing of the T cell Receptor Beta chain from sorted CD4+CD161+ or CD161- T cell clones and analysis was performed by iRepertoire.

### Single-cell RNA sequencing and data analysis

Single-cell OPSCC tumor digests were analyzed by single-cell RNA sequencing (Abdulrahman *et al* submitted), see online supplemental material. External Smart-seq2 datasets of T cells from non-small cell lung cancer (NSCLC),^24^ colorectal cancer (CRC)^25^ and hepatocellular carcinoma (HCC)^26^ were obtained from EGA under accession numbers EGAS00001002430, EGAD00001003910 and EGAS00001002072, respectively. Raw FASTQ files were processed using the nf-core Smart-seq2 pipeline (https://nf-co.re/smartseq2). Read counts were loaded into scanpy and filtered for cells with >2000 detected genes, and <16% mitochondrial reads. The cell-type labels from the original publications were used. A Nextflow pipeline^27^ to reproduce the analyses is publicly available from GitHub (https://github.com/icbi-lab/duurland2021_paper).

### Statistical analysis

Statistical analysis was performed using GraphPad Prism V.9.0.1. For survival analysis, patients were grouped into high and low numbers of cell population based on the median level in all patients combined and statistical significance was analyzed by log-rank testing. Non-parametric (Wilcoxon matched-pairs signed rank test or Mann-Whitney with Holm-Šídák multiple comparison test), one-way analysis of variance (ANOVA) Kruskal-Wallis with Dunn’s multiple comparison test, and RM Two-Way ANOVA or mixed model in case of missing values with Geisser-Greenhouse correction with Tukey’s or Šídák’s multiple comparisons test were performed as appropriate. Where used, lines or bar graphs represent mean or median, and error bars represent standard deviation (SD) or standard error of the mean (SEM) as indicated in figure legends. P values below 0.05 were considered significant and are shown in graphs as *p<0.05, **p<0.01, ***p<0.001, ****p<0.0001.

## Results

### Tumor-infiltrating CD4+CD161+ Tem are associated with improved survival

**Figure 1:**
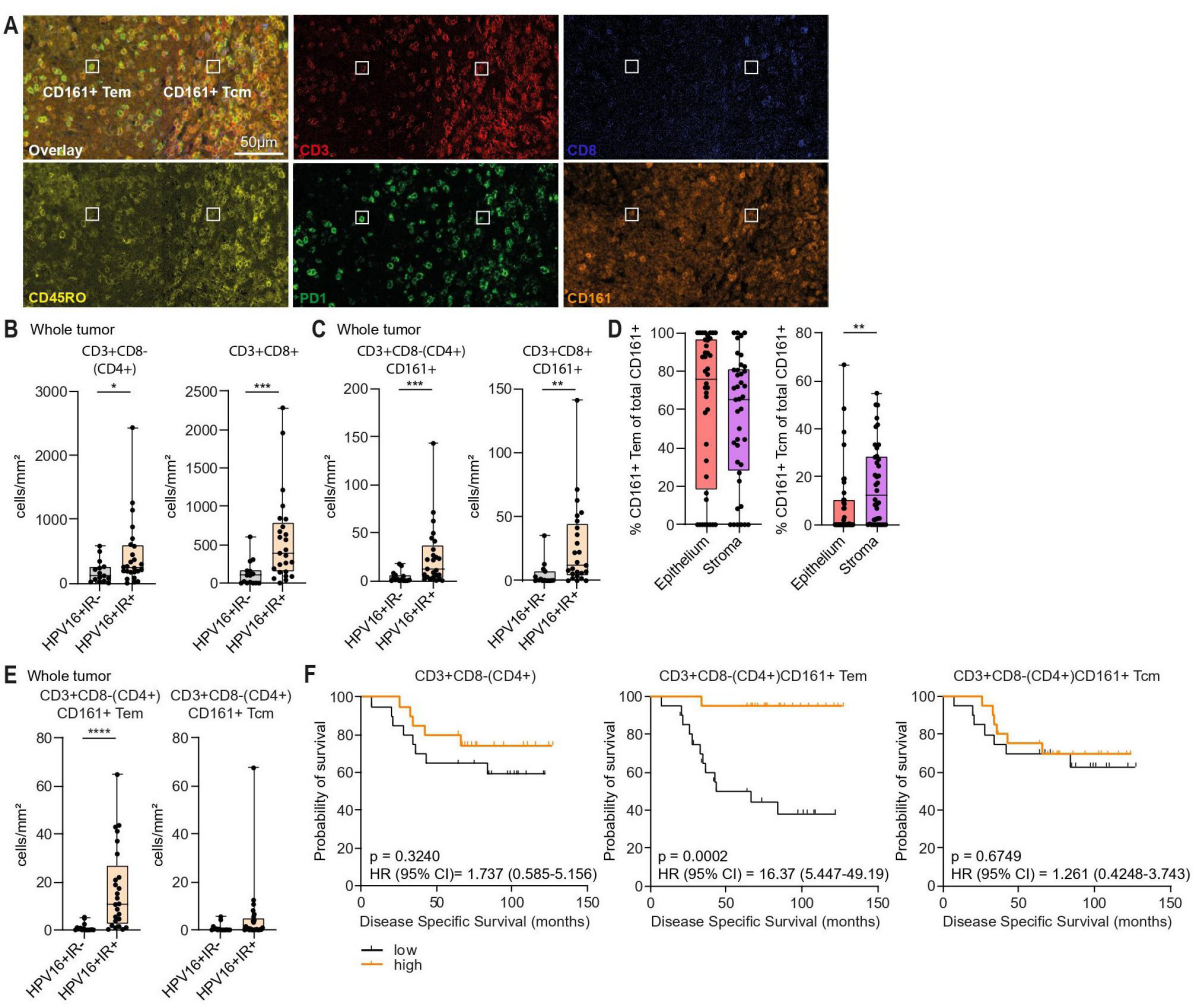
Tumor-infiltrating CD4+CD161+ Tem are associated with improved survival. Formalin-fixed paraffin-embedded (FFPE) sections of OPSCC tumor tissue from HPV16+IR- (n=15) and HPV16+IR+ (n=25) patients were analyzed by immunofluorescence with antibodies against CD3, CD8, CD161, CD45RO, PD1. (A) Overlay and single stains of DAPI, CD3, CD8, CD45RO, PD1, CD161 immunofluorescence staining in a representative HPV16+IR+ sample. White squares indicate CD4+CD161+ Tem and Tcm cells. (B) Summary graphs showing number of CD3+CD8-(CD4+) and CD3+CD8+ T cells/mm^2^ in tumor tissue of HPV16+IR- and HPV16+IR+ patients. (C) Summary graphs showing number of CD3+CD8-(CD4+)CD161+ and CD3+CD8+CD161+ cells/mm^2^ in tumor tissue of HPV16+IR- and HPV16+IR+ patients. (D) Summary graph showing percentage CD161+ Tem and Tcm of total CD161+ T cells in epithelium and stroma. (E) Summary graphs showing cells/mm^2^ for CD3+CD8-(CD4+)CD161+ Tem and CD3+CD8-(CD4+)CD161+ Tcm in HPV16+IR- and HPV16+IR+ patients. (F) Kaplan-Meier survival curves showing survival of patients divided into below (low) or above (high) median numbers of CD3+CD8-(CD4+) cells, CD3+CD8-(CD4+)CD161+ Tem and CD3+CD8-(CD4+)CD161+ Tcm. *p<0.05, **p<0.01, ***p<0.001, ****p<0.0001. HPV16, human papillomavirus 16; IR+/-, immune response positive/negative; Tcm, central memory T cells; Tem, effector memory T cells.

To determine the relevance of CD161 expressing T cells, the TME of 40 OPSCC patients was analyzed using multispectral immunofluorescence (figure 1A). The numbers of total CD3+CD8- (CD4+) (99% of CD3+CD8- T cells are CD4+^19^), CD3+CD8+ cells (figure 1B), CD4+CD161+ and CD8+CD161+ cells (figure 1C) were significantly higher in HPV16+ immune response positive (IR+) compared with HPV16+ IR negative (IR-) patients. Our previously published mass cytometry analysis demonstrated an enrichment of CD4+CD161+ Tem, but not CD4+CD161+ central memory T cells (Tcm), in the TME of HPV16+IR+ patients.^2 3^ Reanalysis of this data^2^ showed that distinct expression patterns of CD45RO and PD1 between Tem and Tcm allowed for discrimination between CD161+ Tem and Tcm (online supplemental figure 1A-B). The percentage CD161+ Tem was similar between epithelium and stroma, whereas the percentage CD161+ Tcm was higher in stroma compared to epithelium (figure 1D). The TME of HPV16+IR+ patients showed a significant enrichment of CD4+CD161+ Tem, but not Tcm, compared to HPV16+IR- patients (figure 1E). For CD8+ cells, numbers of tumor-infiltrating CD161+ Tem and Tcm were higher in HPV16+IR+ compared to HPV16+IR- (online supplemental figure 1C). Numbers of tumor-infiltrating CD4+ cells were not associated with disease-specific survival (DSS), but subgrouping patients based on CD4+CD161+ Tem numbers resulted in a clear separation of survival curves with increased DSS for the group of patients with high CD4+CD161+ Tem levels. This was not the case when patients were grouped by CD4+CD161+ Tcm numbers (figure 1F). Similar analysis for CD3+CD8+ cell populations showed no additional improvement of DSS when CD8+CD161+ Tem or Tcm were grouped compared to total CD8+ cells (online supplemental figure 1D). Thus, specifically CD4+CD161+ Tem are associated with better clinical outcome in OPSCC.

### Type 1 cytokine-producing CD4+CD161+ T cells are expanded upon vaccination

**Figure 2:**
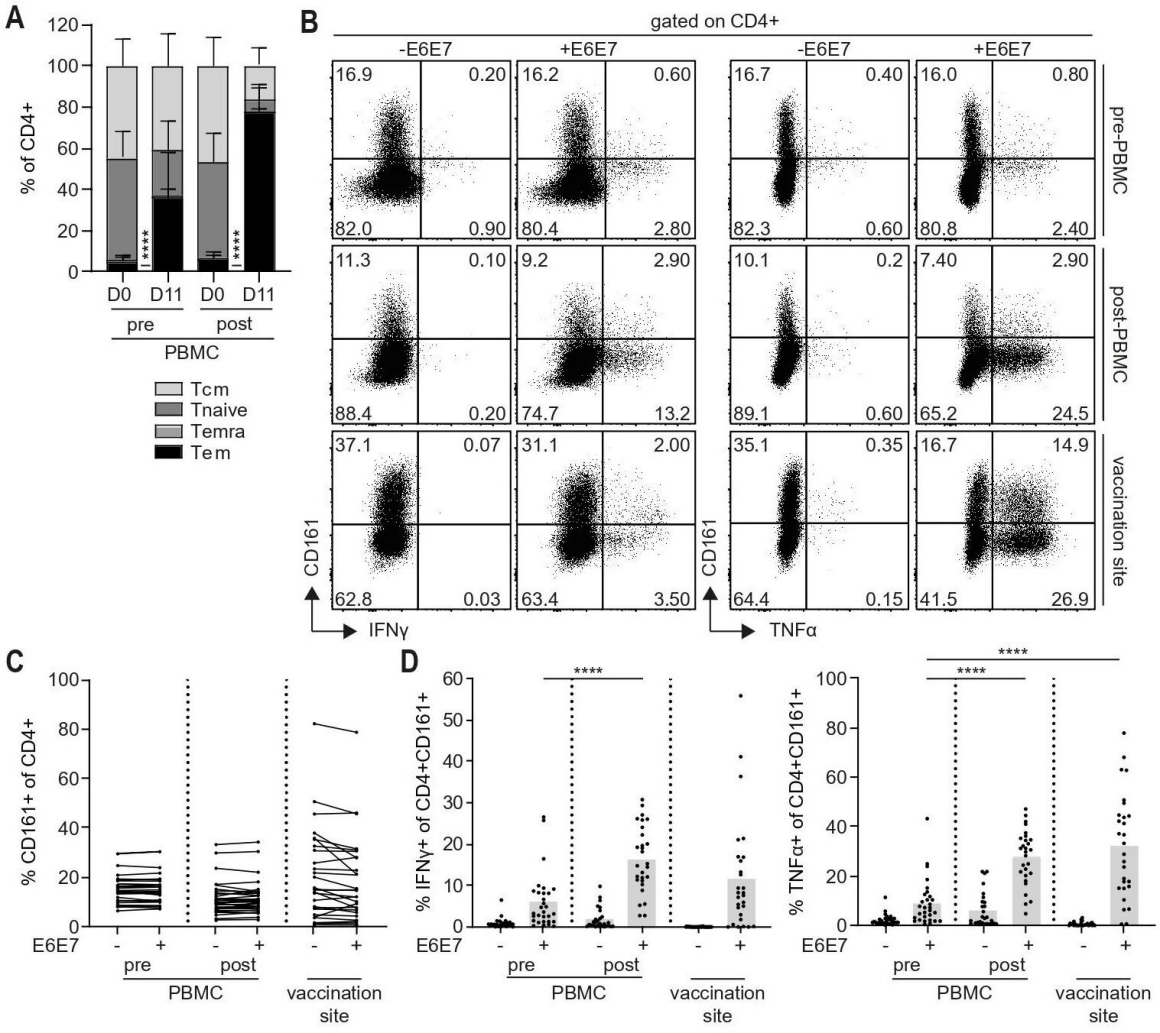
Type 1 cytokine-producing CD4+CD161+ T cells are expanded upon vaccination. (A) PBMC prevaccination (n=28) and postvaccination(n=30) (pre-PBMC and post-PBMC) were prestimulated with a pool of E6E7 peptides (2.5 µg/mL of each peptide) for 11 days to generate bulk cultures. Summary graph depicting percentages Tcm, Tnaive, Temra and Tem of CD4+ cells among pre-PBMC and post-PBMC on day 0 (D0) and after 11 days (D11) prestimulation with E6E7 peptide pool. Graph shows mean±SD. (B, C) Prestimulated pre-PBMC (n=28) and post-PBMC (n=30), and cultured T cells from the vaccination site (n=28) were stimulated overnight with autologous monocytes loaded with a pool of E6E7 peptides (5 µg/mL of each peptide) and analyzed by intracellular cytokine staining. (B) Representative dot plots showing IFNγ and TNFα against CD161 gated on CD4+ cells. (C) Summary graph showing percentage CD161+ cells of CD4+ cells upon stimulation with or without E6E7 peptide pool. (D) Summary graph showing percentage IFNγ+ and TNFα+ of CD4+CD161+ cells upon stimulation with or without E6E7 peptide pool. Bar graphs show mean. ****p<0.0001. IFNγ, interferon γ; PBMC, peripheral blood mononuclear cells; Tcm, central memory T cells; Tem; effector memory T cells; TNFα, tumor necrosis factor α.

The prognostic impact of CD4+CD161+ Tem provides rationale for the investigation of strategies to boost this population. To explore this, biological samples from patients treated with a clinically effective therapeutic HPV16 vaccine^20 21^ were used. Prevaccination and postvaccination PBMC (pre- and post-PBMC), prestimulated *in vitro* with a pool of HPV16 E6E7 peptides to expand HPV16-specific T cells, and T cells cultured from skin biopsies taken from the vaccination site were examined for presence of HPV16-specific responses by CD4+CD161+ cells. Stimulation with E6E7 peptides resulted in detection of IFNγ+, tumor necrosis factor (TNF)α+ and activated (CD154+/CD137+ single and double positive (SP and DP)) CD4+ cells (online supplemental figure 2A–C), which were increased in peripheral blood after vaccination and present at the vaccination site. *In vitro* expansion of pre-PMBC and post-PMBC induced a shift in memory phenotype of CD4+ cells and cells adopted a Tem phenotype, especially postvaccination (figure 2A), but total percentages of CD4+CD161+ cells were not altered (online supplemental figure 2D). Both CD4+CD161+ and CD4+CD161- cell populations contained HPV16-specific T cells producing IFNγ and TNFα upon antigen-specific stimulation (figure 2B–D; online supplemental figure 2E). Furthermore, their frequencies were increased after vaccination (figure 2D) indicating that cytokine-producing CD4+CD161+ cells are boosted by therapeutic vaccination.

### CD161 does not affect cytokine production by CD4+ T cells

**Figure 3:**
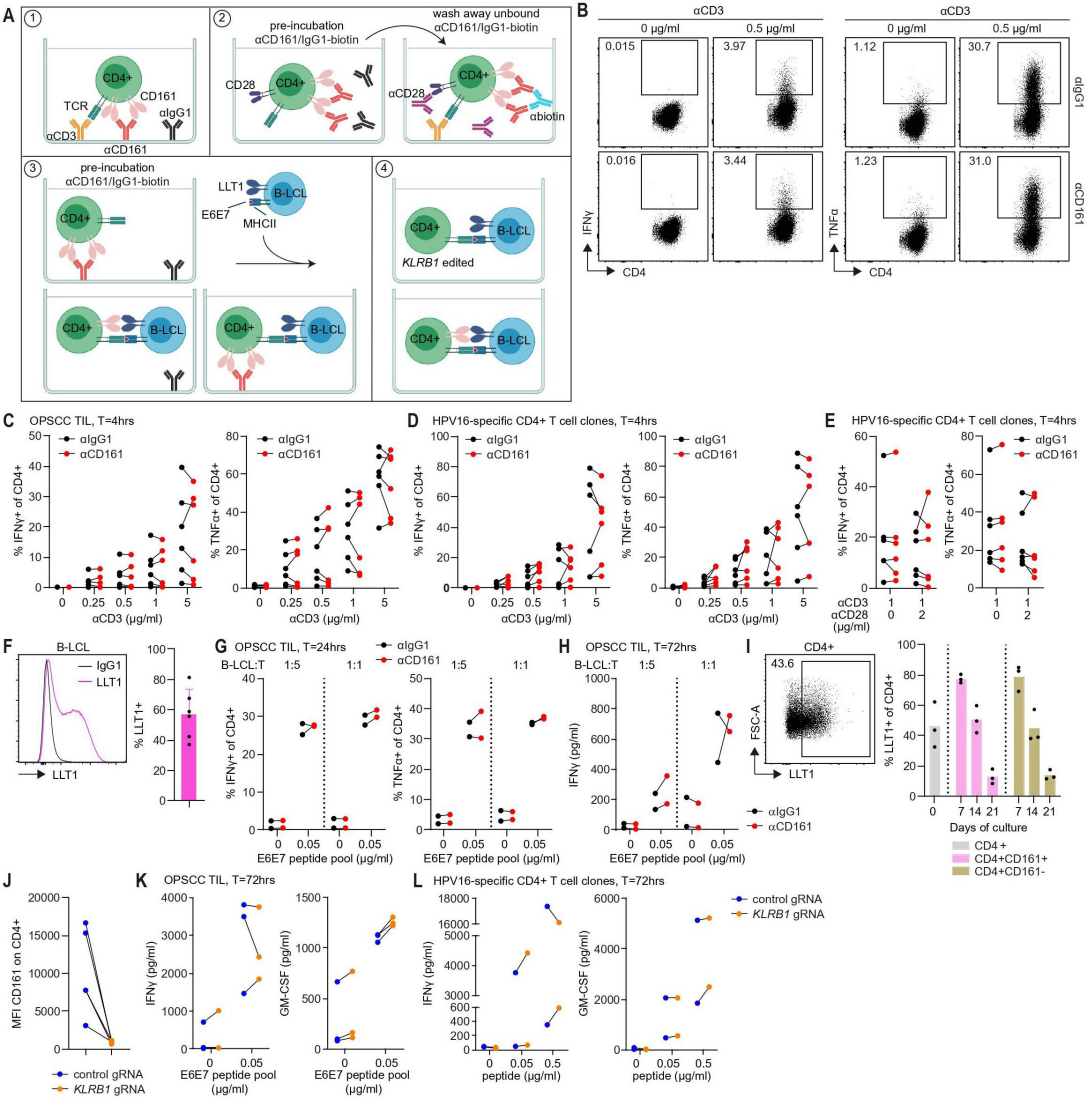
CD161 does not function as a checkpoint molecule on CD4+ T cells. (A) Graphical illustration of the four experimental approaches used to study the function of CD161. (#1) Cells were stimulated with plate-bound anti-CD3 (αCD3, clone UCHL1, indicated concentrations) and plate-bound anti-IgG1 (αIgG1, clone MOPC-21) or anti-CD161 (αCD161, clone HP-3G10) (OPSCC TIL: 10 µg/mL, HPV16-specific CD4+ T cell clones: 5 µg/mL) for 4 hours. (#2) Cells were preincubated with biotinylated anti-IgG1 (clone MOPC-21) or anti-CD161 (clone HP3G10) (5 µg/mL) for 30 min, unbound antibodies were washed away followed by crosslinking with anti-biotin antibody during CD3-mediated TCR stimulation (plate-bound, 1 µg/mL) and CD28 (αCD28, clone CD28.2, 2 µg/mL) costimulation for 4 hours. (#3) Cells were preincubated with plate-bound anti-IgG1 or anti-CD161 (10 µg/mL) before peptide-loaded LLT1 expressing B-LCL were added at a ratio B-LCL:T cell of 1:5 and 1:1 for 24 and 72 hours. (#4) Control or *KLRB1* edited cells (*KLRB1* KO) were cocultured with peptide-loaded LLT1 expressing B-LCL at a ratio B-LCL:T cell of 1:1 for 72 hours. This illustration was created with BioRender.com. (B–D) Representative dot plots and summary graphs showing percentage IFNγ+ and TNFα+ of CD4+ cells upon stimulation of CD4+CD161+ TIL (B-C, n=6) and HPV16-specific CD4+ T cell clones (D, n=6) using approach 1. (E) Graphs show percentages IFNγ+ and TNFα+ of CD4+ cells upon stimulation using approach 2 (n=6). (F) Histogram overlay showing LLT1 staining and summary graph showing percentage LLT1+ cells on B-LCL generated from HPV16+IR+ OPSCC and cervical cancer patients (n=6). (G) Summary graphs showing percentages IFNγ+ and TNFα+ of CD4+ cells upon stimulation of CD4+CD161+ TIL for 24 hours using approach 3 (n=2). (H) IFNγ production upon stimulation of CD4+CD161+ TIL for 72 hours using approach 3 (n=2). (I) Representative dot plot and summary graph showing percentage LLT1+ of CD4+ cells from OPSCC TIL on day 0 and during expansion culture of sorted CD4+CD161+ and CD161- cells (n=3). Bar graph shows mean. (J) Summary graph showing depletion of CD161 protein expression on CD4+ cells in *KLRB1* edited cells compared to control (n=5). (K–L) IFNγ and GM-CSF production by *KLRB1* edited CD4+CD161+ TIL (K, n=3) and HPV16-specific CD4+ T cell clones (L, n=2) upon stimulation using approach 4. B-LCL, B lymphoblastoid cell lines; HPV16, human papillomavirus 16; IFNγ, interferon γ; IR+, immune response positive; LLT1, Lectin-like transcript 1; OPSCC, oropharyngeal squamous cell carcinoma; TCR, T cell receptor; TIL, tumor-infiltrating lymphocytes; TNFα, tumor necrosis factor α.

The effect of the interaction between CD161 and its ligand LLT1 on T cell function is unclear and studies used distinct experimental approaches to examine this.^8 10 12 13 15–17^ The function of CD161 was analyzed using *in vitro* expanded CD4+CD161+ TIL and CD4+CD161+ T cell clones derived from HPV16+IR+ OPSCC patients according to the approaches depicted in figure 3A. First, cells were stimulated with plate-bound anti-CD3 and anti-IgG1 or anti-CD161. In agreement with previous findings,^13 15 28^ TCR stimulation was required for cells to produce IFNγ and TNFα, but specific CD161 engagement on CD4+CD161+ TIL and clones had no consistent effect on cytokine production compared to isotype control (figure 3B–D). Second, cells were stimulated with biotinylated anti-CD161 or anti-IgG1 followed by crosslinking with an anti-biotin antibody during CD3-mediated TCR stimulation and CD28 costimulation, but again no consistent effect was observed (figure 3E). Incubation with anti-CD161 was efficient as indicated by reduced detection of CD161 (online supplemental figure 3A–D). In the third approach, cells were stimulated with cognate antigen presented by LLT1 expressing target cells to interrogate the function of the CD161-LLT1 interaction in a more physiological antigen-specific setting. LLT1 is expressed on Epstein Barr Virus (EBV)-transformed B-LCL,^12 13^ including those generated from OPSCC and cervical cancer patients (figure 3F). Stimulation with cognate antigen resulted in production of IFNγ and TNFα, but no effect of anti-CD161 was observed after 24 or 72 hours of stimulation (figure 3G–H). CD161 expression could not be detected after stimulation with anti-CD161 indicating that blockade was efficient (online supplemental figure 3E). We observed that activated T cells also express LLT1, which was gradually lost over time (figure 3I). This may complicate CD161 blocking experiments, because LLT1 and CD161 can directly interact with each other on T cells before the antibody does. Recently, a coinhibitory effect of CD161 on antigen-specific CD8+ T cells was demonstrated using T cells in which expression of *KLRB1* was edited for inactivation^10^ (figure 3A). Crispr-Cas9-mediated editing of *KLRB1* in CD4+CD161+ OPSCC TIL and HPV16-specific CD4+CD161+ T cell clones was highly effective (figure 3J, online supplemental figure 3F), but did not consistently affect the response of T cells upon stimulation with peptide-loaded LLT1 expressing B-LCL (figure 3K–L). Thus, in all four approaches, no consistent effect on cytokine production by CD4+ cells was observed, ruling out a role for CD161 as coinhibitory receptor on CD4+ cells.

### CD161 expression is dynamic upon TCR triggering and in time

**Figure 4:**
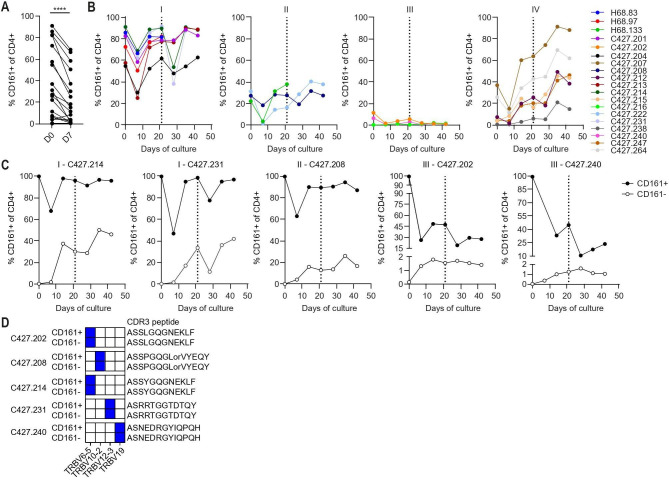
CD161 expression is dynamic upon TCR triggering and in time. HPV16-specific CD4+ T cell clones from HPV16+IR+ OPSCC (n=7) and cervical cancer patients (n=16) were expanded for 3 weeks. (A) Percentage CD161+ of CD4+ cells within CD4+ T cell clones on day 0 and day 7 of culture (n=19). (B) Percentage CD161+ of CD4+ cells during culture of HPV16-specific T cell clones (n=19) divided into 4 groups of CD161 expression patterns: I – >50% CD161+ (n=7), II – 20%–50% CD161+ (n=3), III – <20% CD161+ (n=3), IV – % CD161+ increases over time (n=6). Dotted line indicates re-stimulation of clones with their cognate peptide for another 3 weeks. (C, D) HPV16-specific CD4+ T cell clones (n=5) belonging to group I (n=2), II (n=1) and III (n=2) were sorted into CD161+ and CD161- T cell populations. (C) Sorted clones were cultured and percentage CD161+ T cells was monitored over time. Dotted line indicates re-stimulation of clones with their cognate peptide. (D) TRBV repertoire was determined on day of sort (day 0 of culture) for CD4+CD161+ and CD161- cell populations of each sorted T cell clone using iRepertoire (n=5). ****p<0.0001. HPV16, human papillomavirus 16; IR+, immune response positive; OPSCC, oropharyngeal squamous cell carcinoma; TCR, T cell receptor; TRBV, T cell receptor beta variable.

Analysis of a series of HPV16-specific CD4+ T cell clones with distinct cytokine producing phenotypes (online supplemental figure 4A) revealed a significant reduction in percentage of cells expressing CD161 after 7 days of stimulation (figure 4A). Detailed monitoring of CD161 expression during culture revealed four distinct patterns based on percentage CD161+ cells. Group I showed high percentages of CD161+ cells at start of culture followed by a reduction on day 7 after stimulation, but in time percentages returned to the levels observed at start of culture until the next antigenic stimulation. Group II showed similar kinetics as group I, but percentages of CD161+ cells were intermediate. Percentages of CD161+ cells in group III were low at start of culture and remained low. Group IV followed a distinct pattern as percentages of CD161+ cells were initially reduced after stimulation, but then increased in time (figure 4B). To explore the dynamics of CD161 protein expression, CD161+ and CD161- cells were sorted from HPV16-specific CD4+ T cell clones belonging to group I-III (online supplemental figure 4B). Interestingly, CD161- sorted cells from clones that were at least 25%–30% CD161+ before sorting re-expressed CD161 again in time whereas CD161+ sorted cells remained highly CD161+. The percentage of CD161+ cells sorted from clones which were <20% CD161+ was reduced with time and sorted CD161- cells remained CD161- (figure 4C). Human T cell Receptor Beta Variable (TRBV) gene repertoire analysis of CD4+CD161+ and CD161- cells after cell sorting showed that both cell populations had the same TRBV chain and thus originate from a single T cell clone (figure 4D). These data indicate that CD161 expression is dynamic in time and influenced by TCR-mediated signaling.

### Downregulation of CD161 protein expression depends on TCR-signaling strength

**Figure 5:**
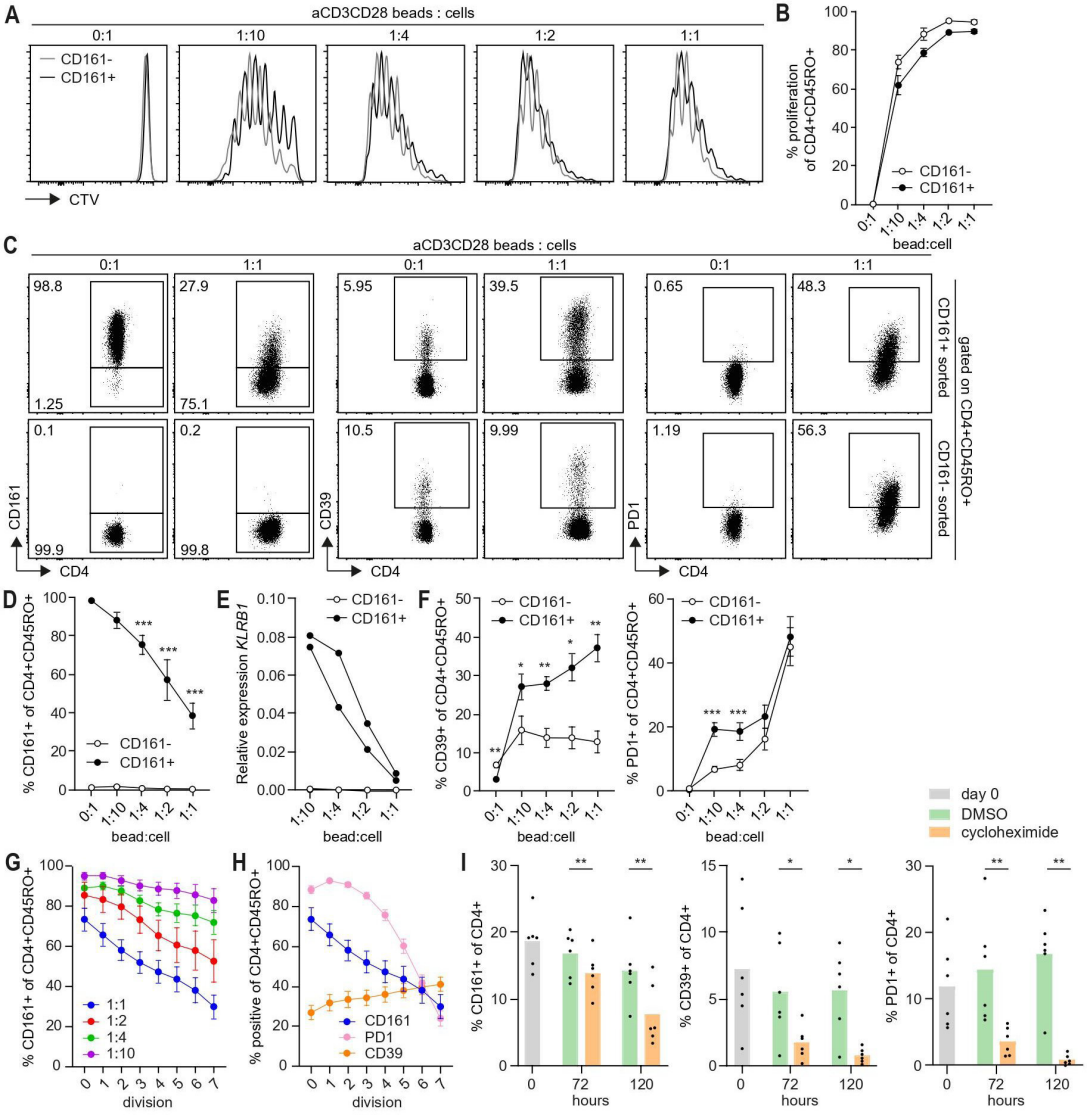
Downregulation of CD161 protein expression depends on TCR-signaling strength. (A–H) CD4+CD161+ and CD161- cells were sorted from PBMC of adult healthy controls, labeled with CellTrace Violet (CTV) and stimulated for 5 days in presence of anti-CD3CD28 beads to cell ratio (bead:cell) as indicated (n=5–9 unless otherwise indicated). Summary data represent mean±SEM. (A) Representative histogram overlays for CD4+CD45RO+CD161+ and CD161-cells showing CTV dilution for anti-CD3CD28 stimulation as indicated. (B) Summary graph showing percentage proliferation of CD4+CD45RO+CD161+ and CD161- cells upon stimulation with anti-CD3CD28. (C) Representative dot plots showing percentage CD161+, CD39+ and PD1+ cells within CD4+CD161+ and CD161- cultures gated on CD4+CD45RO+ cells for anti-CD3CD28 beads to cell ratio as indicated. (D) Summary graph showing percentage CD161+ cells within CD4+CD161+ and CD161- cultures cells gated on CD4+CD45RO+ cells after TCR stimulation. Significance was analyzed for bead:cell of 1:10-1:1 compared to 0:1. (E) Relative expression of *KLRB1* (CD161) within CD4+CD161+ and CD161- cultures after 5 days of TCR stimulation (n=2). (F) Summary graph showing percentage CD39+ and PD1+cells within CD4+CD161+ and CD161- cultures gated on CD4+CD45RO+ cells after TCR stimulation. Significance was analyzed for CD161+ compared to CD161- cells. (G) Percentage CD161+ of CD4+CD45RO+ cells within CD4+CD161+ culture per division after stimulation with anti-CD3CD28 beads to cell ratio as indicated. (H) Percentage CD161+, CD39+ and PD1+ cells within CD161+ cultures gated on CD4+CD45RO+ cells per division for anti-CD3CD28 beads to cell ratio of 1:1. (I) CD4+ cells from adult healthy controls were cultured in presence of 20 µg/mL cycloheximide (CHX) or DMSO vehicle control for 72 and 120 hours and analyzed for percentage CD161+, CD39+ and PD1+ of CD4+ cells (n=6). Summary data represent mean±SEM. *p<0.05, **p<0.01, ***p<0.001. DMSO, dimethyl sulfoxide; *KLRB1*, killer cell lectin-like receptor B1; PBMC, peripheral blood mononuclear cells; TCR, T cell receptor.

To further decipher the effect of TCR stimulation on CD161 expression, CD4+CD161+ and CD161- cells were sorted from healthy donor-derived PBMC (online supplemental figure 5A), stimulated with anti-CD3CD28 beads at different ratio’s and analyzed after 5 days of culture based on the kinetics of expression on T cell clones. We focused on CD4+CD45RO+ cells as circulating CD4+CD161+ cells are mainly CD45RO+ memory cells while CD4+CD161- cells are a mixture of CD45RO+ and CD45RO- cells^6^ which become CD45RO+ after anti-CD3CD28 stimulation (online supplemental figure 5B). The proliferative capacity of CD4+ CD45RO+CD161+ and CD161- cells after TCR stimulation was similar (figure 5A–B). TCR stimulation resulted in a dose-dependent decrease in percentage CD161+ cells and CD161 expression per cell (figure 5C–D, online supplemental figure 5C). This was due to decreased transcription as *KLRB1* levels were strongly declined at day 5 (figure 5E). In contrast to CD161, the percentages of cells expressing the coinhibitory molecules PD1 and CD39 were increased after TCR stimulation and especially CD161+ sorted cell cultures contained higher percentages of CD39+ and PD1+ cells (figure 5C, F, online supplemental figure 5D). Furthermore, the percentage of stimulated cells expressing CD161 declined with each cell division at a linear rate (figure 5G–H). In contrast, the percentage PD1+ cells remained high for two divisions, but rapidly declined thereafter whereas the percentage CD39+ cells slowly increased with each division round within CD161+ sorted cells (figure 5H). The strong decline in CD161/*KLRB1* expression upon TCR stimulation and linear reduction in CD161 cell surface expression with cell division is different from the kinetics observed for PD1 and CD39 suggesting differences in cell surface protein stability. Therefore, CD4+ cells were cultured in presence of cycloheximide (CHX), which blocks protein translation, and expression of the different proteins was followed in time. While percentages of PD1+ and CD39+ cells were strongly reduced within 72 hours, the decline in percentage of CD161+ cells was much slower with only 50% reduction after 120 hours of culture (figure 5I). Thus, expression of CD161 is downregulated by TCR signaling at both mRNA and protein level, and its expression pattern is opposite to the known coinhibitory markers PD1 and CD39. This is consistent with our observation that CD161 does not function as a coinhibitory molecule in CD4+ cells.

### TGFβ1 regulates CD161 expression on CD4+ T cells

**Figure 6:**
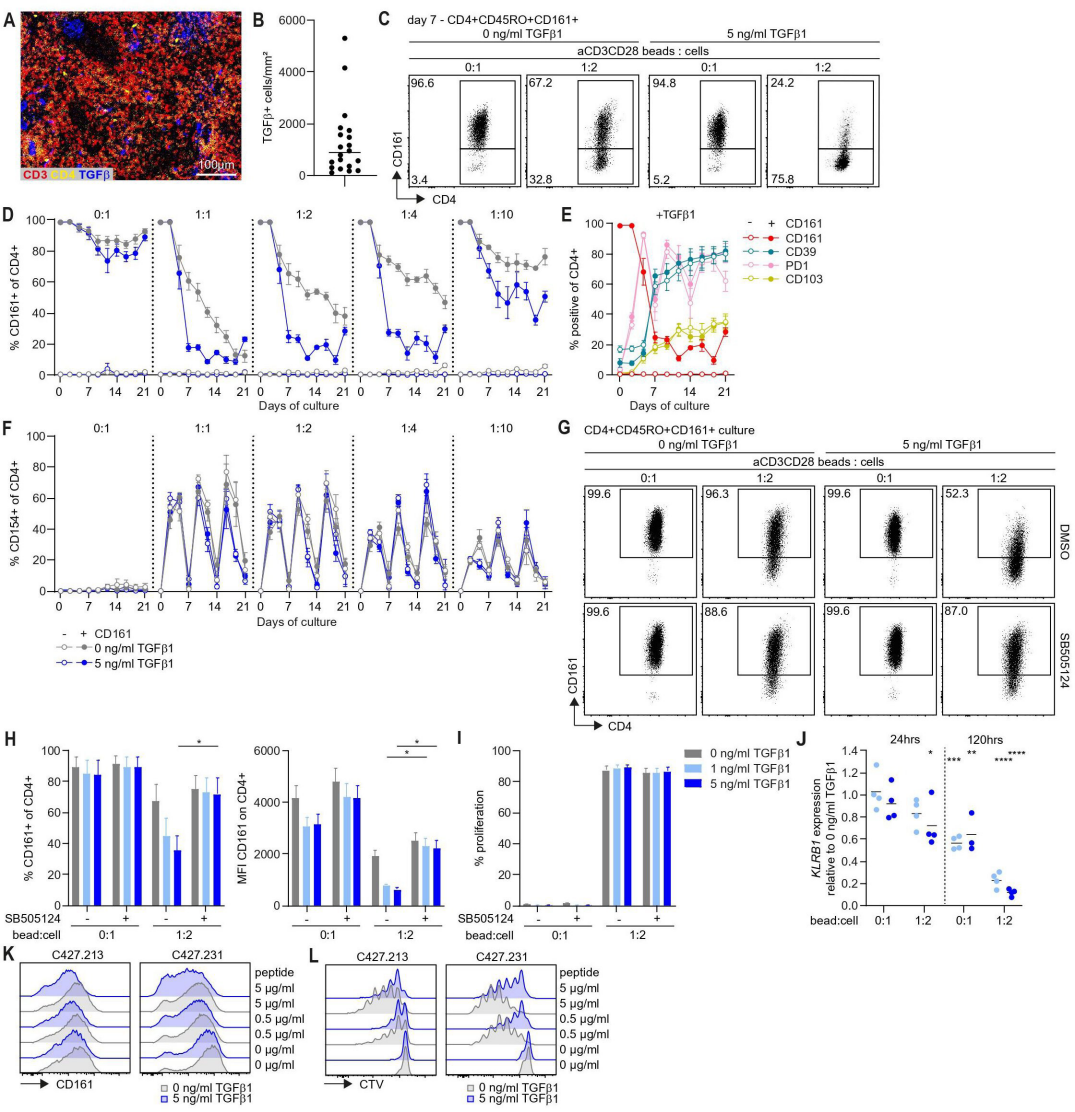
TGFβ1 reduces CD161 expression on CD4+ T cells. (A) FFPE tumor tissue of OPSCC patients was analyzed by imaging mass cytometry for CD3 (red), CD4 (yellow) and TGFβ (blue) expression. A representative example of staining in a HPV16+IR+ sample is shown. (B) TGFβ+ cells/mm^2^ in tumor tissue sections of OPSCC patients as determined by imaging mass cytometry. Line indicates median. (C–F) CD4+CD45RO+CD161+ and CD161- cells were sorted from PBMC of adult healthy controls and cultured in presence of 0 or 5 ng/mL TGFβ1, 5 ng/mL rhIL-7 and anti-CD3CD28 beads at bead to cell ratio as indicated (n=3–5). Cells were restimulated with anti-CD3CD28 beads every 7 days and medium containing TGFβ1 and rhIL-7 was refreshed every 3–4 days. Graphs show mean±SEM. (C) Representative dot plots showing percentage CD161+ of CD4+ cells within the CD4+CD161+ culture in presence or absence of TGFβ1 and anti-CD3CD28 beads as indicated on day 7 of culture. (D) Percentage CD161+ of CD4+ cells within CD4+CD161+ and CD161- cultures in time. (E) Percentage CD161+, CD39+, PD1+, CD103+ of CD4+ cells upon stimulation with anti-CD3CD28 beads to cell ratio of 1:2 and 5 ng/mL TGFβ1. (F) Percentage CD154+ of CD4+ cells within CD4+CD161+ and CD161- cultures in time. (G–J) CD4+CD45RO+CD161+ cells were sorted from PBMC of adult healthy controls, labeled with CTV and cultured in presence of 0,1 or 5 ng/mL TGFβ1, 5 ng/mL rhIL-7, and anti-CD3CD28 beads at bead to cell ratio of 0:1 and 1:2 with or without 1µM SB505124 or DMSO control for 5 days as indicated (n=5 unless otherwise indicated). (G) Representative dots plots showing percentage CD161+ cells within CD4+CD161+ cultures. (H, I) Percentage CD161+ of CD4+ cells, MFI CD161 on CD4+ cells (H), and percentage proliferation (I) within CD4+CD161+ cultures. Graphs show mean±SEM. (J) *KLRB1* expression in CD4+CD161+ cultures stimulated with 1 and 5 ng/mL TGFβ1 relative to 0 ng/mL TGFβ1 with or without anti-CD3CD28 beads after 24 hours and 120 hours (n=3–4). Lines indicate mean and significance was analyzed compared to 0 ng/mL TGFβ1. (K, L) Two HPV16-specific CD4+ T cell clones were labeled with CTV and stimulated with 5 ng/mL rhIL-15, peptide-loaded B-LCL with or without 5 ng/mL TGFβ1 for 5 days. Histograms show CD161 protein expression (K) and CTV dilution (L) for indicated stimuli. *p<0.05, **p<0.01, ***p<0.001, ****p<0.0001. B-LCL, B lymphoblastoid cell lines; CTV, CellTrace Violet; FFPE, formalin-fixed paraffin-embedded; HPV16, human papillomavirus 16; IR+, immune response positive; *KLRB1*, killer cell lectin-like receptor B1; OPSCC, oropharyngeal squamous cell carcinoma; MFI, median fluorescence intensity; PBMC, peripheral blood mononuclear cells; rhIL-7, recombinant human interleukin-7; rhIL-15, recombinant human interleukin-15; TGFβ1, transforming growth factor β1.

Transforming growth factor beta (TGFβ) expressing cells are present at variable levels in OPSCC (figure 6A–B). TGFβ is related to worse overall survival in OPSCC^29^ and known to increase expression of PD1, CD39 and CD103^30 31^ on T cells. To determine if TGFβ affects CD161 expression, healthy donor-derived CD4+CD45RO+CD161+ and CD161- cells were stimulated using anti-CD3CD28 beads in presence or absence of TGFβ1. An increased percentage of CD39+ and PD1+ cells was observed from day 7 of culture in the presence of anti-CD3CD28 stimulation and TGFβ1. The percentage of CD103+ cells was increased from 3 days onwards. This effect was independent of CD161 status (online supplemental figure 6A-B). In contrast, percentages of CD161+ cells decreased when TGFβ1 was present (figure 6C–E). TGFβ1 had no effect on T cell activation as indicated by CD154 expression (figure 6F, online supplemental figure 6C). The observed effect was TGFβ1 specific as it was reversed by addition of SB505124, a selective inhibitor of TGFβ type I receptor (like) kineases, i.e. activin receptor-like kinase (ALK)4, ALK5 and ALK7 signaling, to sorted CD4+CD45RO+CD161+ cells (figure 6G–H, online supplemental figure 6D) or addition of a pan-neutralizing TGFβ antibody to CD4+ cells (online supplemental figure 7A-B). Notably, proliferation of cells was not affected by TGFβ1 or SB505124 (figure 6I). The effect of TGFβ1 tapped into regulation of CD161 at gene level as *KLRB1* expression was decreased after 5 days to about 60% without anti-CD3CD28 stimulation and 80%–90% with anti-CD3CD28 stimulation (figure 6J). We confirmed the effect of TGFβ1 on CD161, PD1 and CD103 for HPV16-specific CD4+CD161+ T cell clones stimulated with different doses of cognate antigen (figure 6K, online supplemental figure 6E). However, TGFβ1 reduced proliferation and the effect on CD39 was minimal because expression levels were already high (figure 6L, online supplemental figure 6E).

Other members of the TGFβ family, such as Activin A, and bone morphogenetic protein (BMP)2 and BMP6 that signal via distinct surface receptors, had no effect on CD161 indicating that the observed effect is specific for TGFβ1. Activin A did increase the percentages of CD39+, PD1+ and CD103+ cells, consistent with published data for CD39 and PD1,^32 33^ and the effect was reversed by SB505124 (online supplemental figure 8).

Thus, the effect of TGFβ1 on expression of CD161 is different from the effect on well-known coinhibitory molecules and not part of general T cell suppression, because activation and proliferation of cells was not affected.

### CD4+CD161+ cells specifically express the transcriptional transactivator *SOX4*

**Figure 7:**
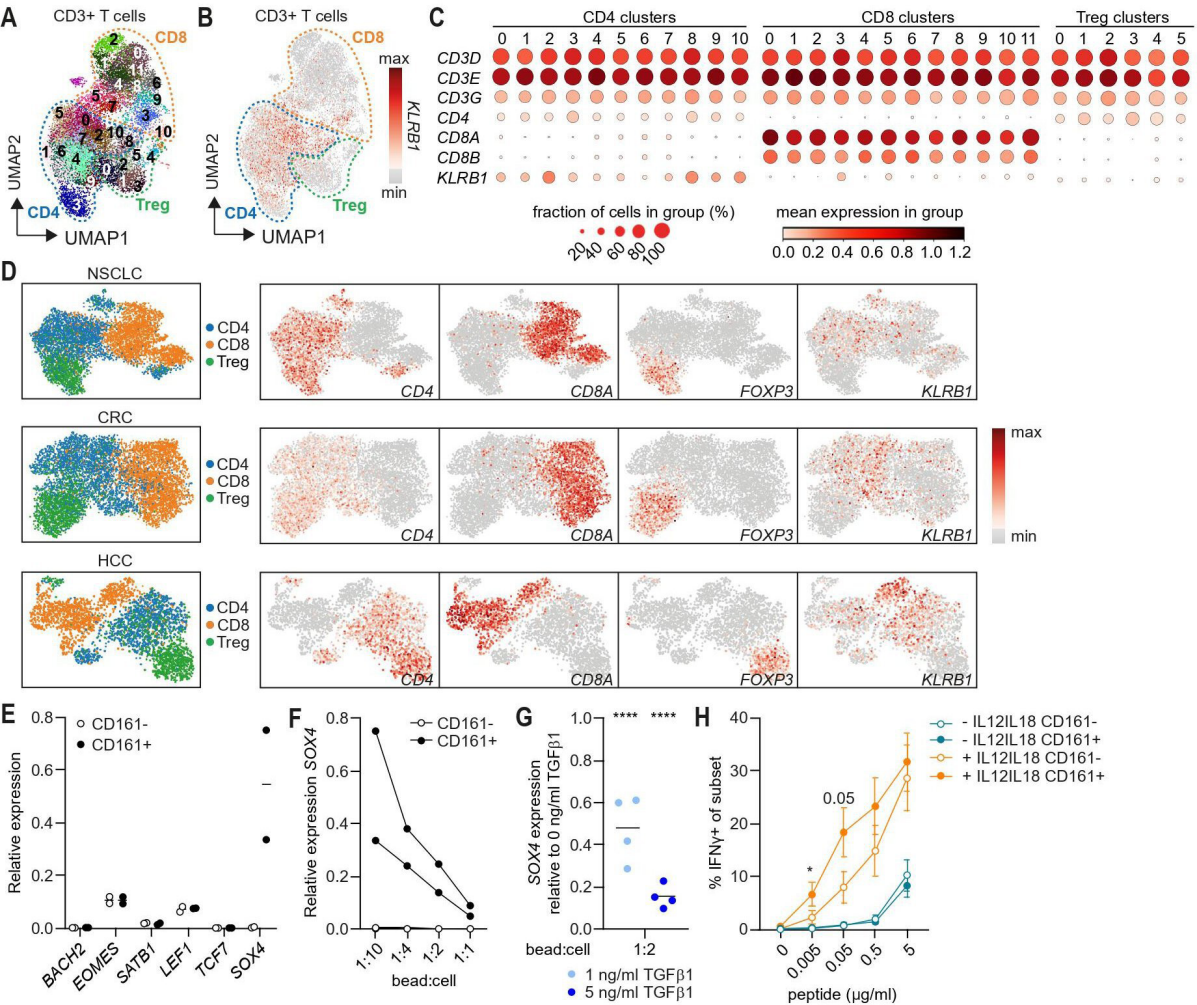
CD161+CD4+ T cells specifically express the transcriptional transactivator *SOX4*. (A–C) *Ex vivo* TIL samples from OPSSC patients were enriched for CD3+CD56+ cells and analyzed by single-cell RNAseq (n=13). Clustering analysis was restricted to CD3+ cells. (A) Two-dimensional UMAP plot showing 29 different CD3+ clusters identified using the Leiden algorithm. These CD3+ clusters could be further divided into 11 CD4+, 12 CD8+ and 6 Treg clusters. (B) Two-dimensional UMAP plot showing distribution of *KLRB1* expression. (C) Plot showing fraction of cells in group (%) and mean expression in group for *CD3D*, *CD3E*, *CD3G*, *CD4*, *CD8A*, *CD8B* and *KLRB1*. (D) Single-cell RNAseq analysis of published datasets for NSCLC,^24^ CRC,^25^ HCC^26^ showing CD4, CD8 and Treg clusters (left) and distribution of *CD4*, *CD8A*, *FOXP3*, *KLRB1* (CD161) expression (right). (E, F) CD4+CD161+ and CD161- cells were sorted from PBMC of adult healthy controls and stimulated for 5 days in presence of anti-CD3CD28 beads to cell ratio (bead:cell) as indicated (n=2). (E) Summary graph showing relative expression of *BACH2*, *EOMES*, *SATB1*, *LEF1*, *TCF7* and *SOX4* upon stimulation of CD4+CD161+ and CD161- cells with bead:cell ratio of 1:10. Line indicates mean. (F) Summary graph showing relative expression of *SOX4* within CD4+CD161+ and CD161- cultures after 5 days of stimulation with anti-CD3CD28 beads. (G) CD4+CD45RO+CD161+ cells were sorted from PBMC of adult healthy controls and cultured in presence of 0,1 or 5 ng/mL TGFβ1 and anti-CD3CD28 beads at bead to cell ratio of 1:2 for 5 days. Graph showing *SOX4* expression relative to 0 ng/mL TGFβ1 after 120 hours (n=4). Lines indicate mean and significance was analyzed compared to 0 ng/mL TGFβ1. (H) Percentage IFNγ+ of CD4+CD161+ or CD161- cells upon stimulation of 17 different HPV16-specific CD4+ T cell clones with peptide-loaded B-LCL with or without 50 ng/mL rhIL-12 and 50 ng/mL rhIL-18 for 24 hours with Brefeldin A added for the last 4 hours. Graph shows mean±SEM. *p<0.05, ****p<0.0001. CRC, colorectal cancer; HCC, hepatocellular carcinoma; *KLRB1,* killer cell lectin-like receptor B1; NSCLC, non-small cell lung cancer; OPSSC, oropharyngeal squamous cell carcinoma; PBMC, peripheral blood mononuclear cells; rhIL-12, recombinant human interleukin-12; rhIL-18, recombinant human interleukin-18; TGFβ1, transforming growth factor β1; TIL, tumor infiltrating lymphocytes; UMAP, uniform manifold approximation and projection.

The observation that CD161 expression is dynamically regulated at gene level after TCR signaling (figure 5E) and expressed on CD4+ cells with different cytokine patterns suggests that CD161 expression by CD4+ cells is likely to reflect an activation state of cells^34^ in human tumors. This would imply that CD161 expression can be found among different T cell clusters in tumors and is associated with expression of particular transcription factors. Unsupervised clustering of CD3+ cells from our single-cell RNA sequencing data of OPSCC patients^35^ (Abdulrahman *et al* submitted) identified 29 different CD3+ clusters of which 11 clusters were CD4+ (figure 7A). Analysis of *KLRB1* expression revealed that it is expressed by multiple CD4+ clusters (figure 7B, C). Similar analysis using publicly available single-cell RNAseq datasets for NSCLC,^24^ CRC^25^ and HCC^26^ confirmed our findings (figure 7D).

Transcriptional profiling of CD27-CD161- stage 1 and CD27+CD161+ stage 3 mucosal associated invariant T (MAIT) cells highlighted differential expression of several transcription factors including *BACH2*, *EOMES*, *SATB1*, *LEF1*, *TCF7* (TCF1), *SOX4* and *RUNX3*.^36^ As expected, expression of *BACH2*, a transcriptional repressor of memory/effector cells,^37^ was low after anti-CD3CD28 stimulation of blood-derived CD4+CD161+ and CD161- cells. There was no difference in expression of *EOMES*, involved in type 1 commitment,^38^ and *SATB1*, a regulator of lineage-specific genes,^39^ between CD4+CD161+ and CD161- cells (figure 7E, online supplemental figure 9A). The transcription factors *LEF1*, *TCF7* and *SOX4* are known to function as T cell enhancers.^40 41^ There was no difference in expression of *LEF1* and *TCF7* between CD4+CD161+ and CD161- cells upon TCR stimulation, but *SOX4* was exclusively expressed in CD161+ cells (figure 7E, online supplemental figure 9A). In contrast to CD4+CD161+ cells, not all CD4+CD161- cells display a memory phenotype after stimulation (online supplemental figure 5B). If *SOX4* expression would simply reflect memory T cells, expression would not be absent in CD4+CD161- cells, but detected at lower levels. Similar to our observations for *KLRB1*/CD161, expression of *SOX4* was reduced upon increasing strength of TCR activation (figure 7F). In line with this, exposure to TGFβ1 had no effect on *LEF1* and *TCF7* expression, but further reduced expression of *SOX4* in a dose-dependent manner (figure 7G, online supplemental figure 9B). Thus, the expression of *KLRB1*/CD161 is coregulated with the T cell enhancer *SOX4* by cell intrinsic and extrinsic factors associated with T cell activation.

Based on the coregulation of *KLRB1*/CD161 and the T cell enhancer *SOX4*, we hypothesized that CD161 expression may reflect an enhanced activation state. To examine this, HPV16-specific CD4+ T cell clones were stimulated with cognate antigen in presence of rhIL-12 and rhIL-18, previously shown to act on CD161+ T cells.^8 42^ Our data show that these cytokines improved the reactivity of both CD4+CD161+ and CD161- populations. However, under limiting amounts of antigen, the percentage of T cells producing IFNγ was significantly higher among the CD161+ population compared to their CD161- counterparts (figure 7H). Thus, these data suggest that CD161 expression by CD4+ cells reflects a specific activation state allowing these cells to respond more vigorously.

## Discussion

In this study, we demonstrated that the TME of HPV16+IR+ OPSCC is highly infiltrated by CD4+CD161+ and CD8+CD161+ Tem. While expression of CD161 did not alter the impact of CD8+ cells on survival, there was a clear clinical benefit when tumors were infiltrated with relatively high numbers of CD4+CD161+ Tem. This suggests that mainly CD4+CD161+ cells contribute to the prognostic impact and improved survival associated with high *KLRB1* expression levels in tumors.^2 3 43 44^ CD4+CD161+ cells display a stronger type 1 response to suboptimal antigen stimulation (this study) and produce more cytokines at a per cell basis upon antigen stimulation.^2^ The stronger reactivity of these cells might be due to a concerted action between the transcriptional transactivator SOX4, which transactivates CD3ε,^41^ enhances TCR signaling^45 46^ and is specifically expressed by CD4+CD161+ cells, and CD161, which interacts with acid sphingomyelinase to generate ceramide required for the signaling cascade downstream of CD3.^47^

The role of CD161 as a costimulatory or coinhibitory molecule is highly debated and the outcomes varied depending on the experimental setup used. Many studies examined CD161 function using plate-bound antibodies or beads coated with anti-CD3 and/or anti-CD28 to stimulate T cells, or LLT1+ cell lines.^8 12 13 15–17^ The most compelling evidence for a coinhibitory function of CD161 on CD8+ cells came from a study in which *KLRB1* expression was knocked-out in NY-ESO-1 TCR transduced T cells.^10^ We used HPV16-specific CD4+CD161+ cells in all these experimental approaches, but found no consistent coinhibitory or costimulatory effect. One of the problems with CD161-specific antibody blockade could be that activated T cells express LLT1^12^, hence LLT1 can directly interact with CD161 and thereby prevent antibody binding. We used *KLRB1* edited T cells to overcome this hurdle, but again no costimulatory or coinhibitory role for CD161 on CD4+ cells was found.

In contrast to increased expression of known checkpoint molecules PD1 and CD39 after T cell activation, expression of CD161 is reduced at both mRNA and protein level. While it is known that TGFβ1 induces expression of CD39, PD1 and CD103 on T cells,^30 31^ we show here that costimulation with TGFβ1, but not other TGFβ superfamily members, resulted in an even stronger reduction of CD161 expression. The observed expression pattern of CD161 on CD4+ cells being the opposite of well-known checkpoints also argues against a coinhibitory function of CD161.

We demonstrated that more HPV16-specific CD4+CD161+ cells produced IFNγ after suboptimal antigen and cytokine stimulation compared to their CD161- counterparts. This is consistent with a report showing that CD4+CD161+ TIL in NSCLC are more activated.^43^ These findings suggest that CD161 is a marker of a specific activation state of CD4+ cells in which cells are more prone to produce type 1 cytokines upon low-dose antigen encounter. In this context, expression of the T cell enhancers *LEF1*, *TCF7* (TCF-1) and *SOX4* is of interest. As expected, expression of *LEF1* and *TCF7,* both important for TCRα gene expression, did not differ between CD4+CD161+ and CD161- cells. *SOX4*, which transactivates CD3ε and enhances TCR signaling,^41 45 46^ shows higher expression in CD4+CD161+ cells. Similar to CD161, *SOX4* expression was reduced upon TCR stimulation and TGFβ1 exposure. Although more research is required to understand the exact role of SOX4 and CD161 in CD4+ cells, it seems that both amplify TCR signals via CD3.^45–47^

In conclusion, relatively high levels of CD4+CD161+ Tem are associated with better clinical outcome. These cells respond more vigorously to TCR stimulation under suboptimal conditions fostered by their responsiveness to innate cytokines^8 42^ and expression of TCR-signaling amplifying molecules SOX4 and potentially CD161 itself. Cytokine-producing CD4+CD161+ effector cells can be induced by therapeutic vaccination, but as TGFβ1 downregulates both *KLRB1*/CD161 and *SOX4*, immunotherapeutic strategies may include TGFβ inhibition.

## Data Availability

All data relevant to the study are included in the article or uploaded as online supplemental information. Data are also available from the corresponding author upon reasonable request.
