# Supplementary material for: CD161 expression and regulation defines rapidly responding effector CD4+ T cells associated with improved survival in HPV16-associated tumors
Source: J Immunother Cancer. 2022 Jan 17;10(1):e003995. doi: 10.1136/jitc-2021-003995 (PMC8765066; doi:10.1136/jitc-2021-003995)
Supplement: Supplementary data [file jitc-2021-003995supp001.pdf]

Supplemental material: Supplementary Figures and Tables

CD161 expression and regulation defines rapidly responding effector CD4+ T cells associated with improved survival in HPV16-associated tumors

Chantal L. Duurland[1*], Saskia J. Santegoets[1], Ziena Abdulrahman[1], Nikki M. Loof[1], Gregor Sturm[2], Tom H. Wesselink[3], Ramon Arens[3], Sanne Boekestijn[1], Ilina Ehsan[1], Mariette I.E. van Poelgeest[4], Francesca Finotello[2,5,6], Hubert Hackl[2], Zlatko Trajanoski[2], Peter ten Dijke[7], Veronique M. Braud[8], Marij J.P. Welters[1], Sjoerd H. van der Burg[1*]

[1] Department of Medical Oncology, Oncode Institute, Leiden University Medical Center, Leiden, The Netherlands; [2] Biocenter, Institute of Bioinformatics, Medical University of Innsbruck, Innsbruck, Austria; [3] Department of Immunology, Leiden University Medical Center, Leiden, The Netherlands; [4] Department of Gynecology, Leiden University Medical Center, Leiden, the Netherlands; [5] Institute of Molecular Biology and [6] Digital Science Center (DiSC), University of Innsbruck, Innsbruck, Austria; [7] Department of Cell and Chemical Biology, Oncode Institute, Leiden University Medical Center, Leiden, The Netherlands; [8] Université Côte d'Azur, Centre National de la Recherche Scientifique, Institut de Pharmacologie Moléculaire et Cellulaire, UMR7275, 06560 Valbonne, Sophia Antipolis, France

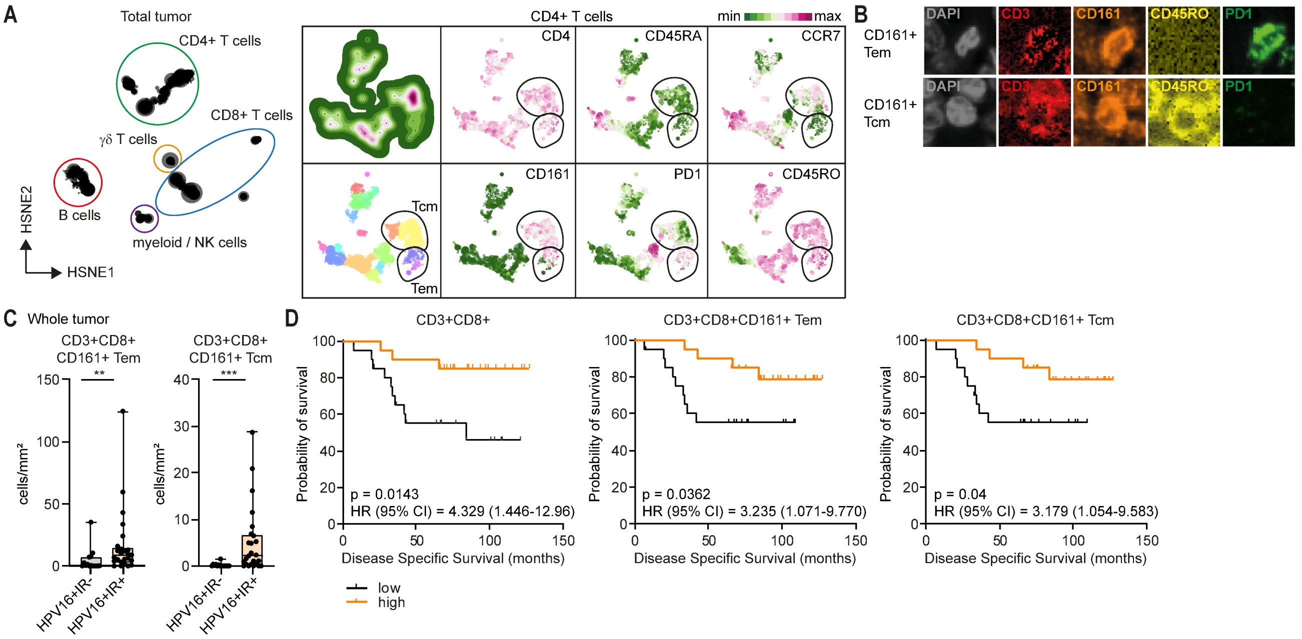

Supplementary Figure 1: CD8+, CD8+CD161+ Tem and CD8+CD161+ Tcm in the TME are associated with improved survival

A) HSNE plot showing different cell populations detected in total tumor and visualization of CD4, CD45RA, CCR7, CD161, PD1, CD45RO expression within CD4+ cells in Tem and Tcm (3). Tem were defined as PD1hiCD45RO+/- (CD45RO+/- were included due to absence of markers in the staining panel that can be used to discriminate between Tem and Tem which re-express CD45RA (Temra)) and Tcm were defined as PD1lowCD45RO+. B-D) FFPE sections of OPSCC tumor tissue from HPV16+IR- (n=15) and HPV16+IR+ (n=25) patients were analyzed by immunofluorescence with antibodies against CD3, CD8, CD161, CD45RO, PD1. B) Close-ups of CD161+ Tem and CD161+ Tcm showing DAPI, CD3, CD161, PD1 and CD45RO staining in a representative HPV16+IR+ sample. C) Summary graphs showing cells/mm² for CD3+CD8+CD161+ Tem and Tcm in HPV16+IR- and HPV16+IR+ patients. D) Kaplan-Meier survival curves showing survival of patients divided into low or high median numbers of CD3+CD8+ cells, CD3+CD8+CD161+ Tem and CD3+CD8+CD161+ Tcm. ** p<0.01, *** p<0.001.

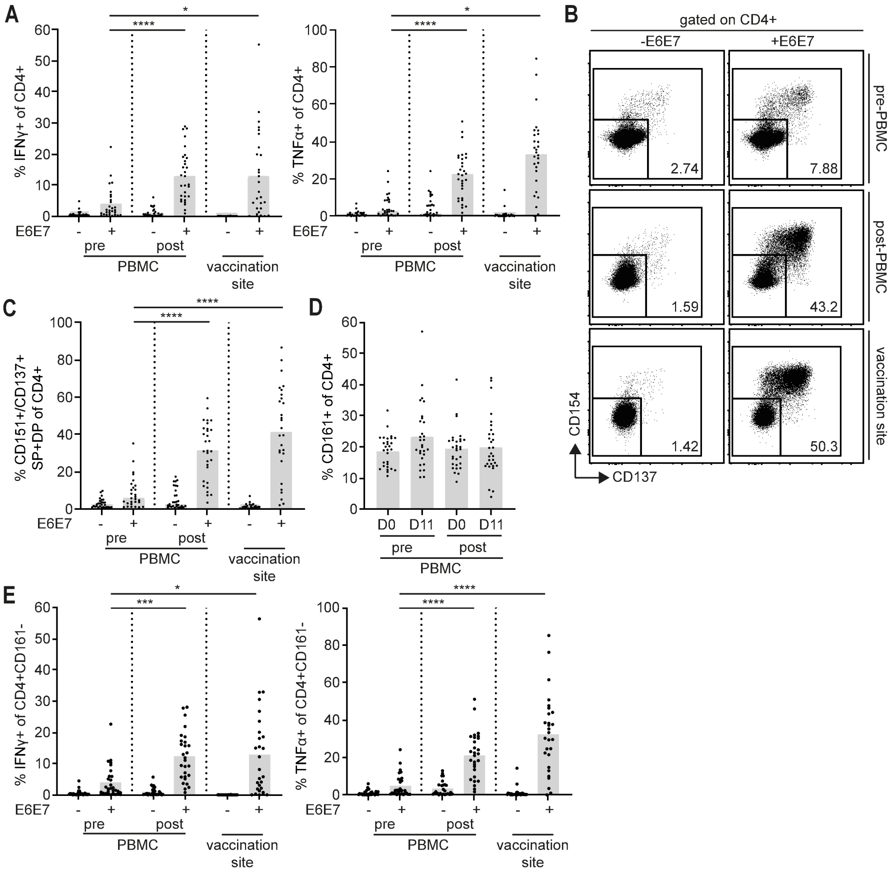

Supplementary Figure 2: Cytokine production by CD4+ cells upon vaccination

PBMC pre- (n=28) and post- (n=30) vaccination (pre- and post-PBMC) were pre-stimulated with a pool of HPV16 E6E7 peptides for 11 days. A-C) Pre-stimulated pre- and post-PBMC and cells from vaccination site (n=28) were stimulated overnight with autologous monocytes loaded with E6E7 peptide pool and analyzed by intracellular cytokine staining. A) Summary graphs showing percentage IFNγ+ and TNF α+ of CD4+ cells upon stimulation with or without E6E7 peptide pool. B-C) Representative dot plots (B) and summary graphs (C) showing percentage CD154+/CD137+ SP+DP of CD4+ cells upon stimulation with or without E6E7 peptide pool. D) Summary graph showing percentage CD161+ of CD4+ cells among pre- and post-PBMC on day 0 (D0) and after 11 days (D11) pre-stimulation with E6E7 peptide pool. E) Summary graphs showing percentage IFNγ+ and TNFα+ of CD4+CD161- cells upon stimulation with or without E6E7 peptide pool. Bar graphs show mean. * $p < 0.05$, *** $p<0.001$, **** $p<0.0001$.

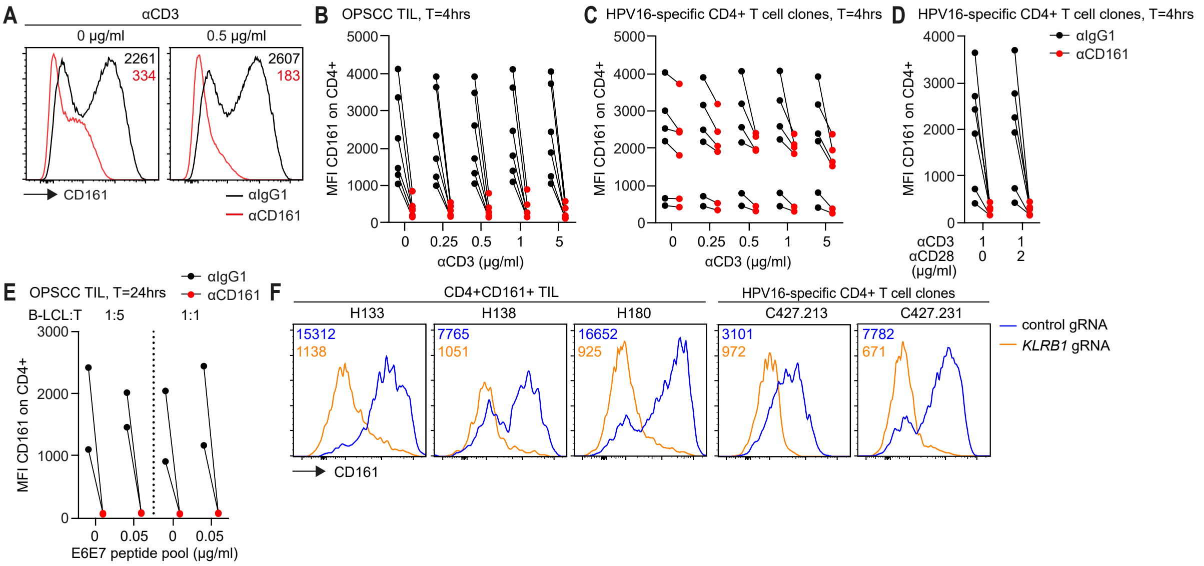

Supplementary Figure 3: Effect of anti-CD161 antibody and KLRB1 editing on CD161 expression

A-C) Representative histogram overlays and summary graphs showing MFI CD161 on CD4+ cells upon stimulation of CD4+CD161+ OPSCC TIL (A-B, n=6) and HPV16-specific CD4+ T cell clones (C, n=6) with plate-bound anti-CD3 (0, 0.25, 0.5, 1, 5 µg/ml) and plate- bound anti-IgG1 or anti-CD161 (OPSCC TIL (A-B): 10 µg/ml, HPV16-specific CD4+ T cell clones (C): 5 µg/ml) for 4 hours. Numbers in overlay indicate MFI of CD161. D) MFI CD161 on CD4+ T cells among HPV16-specific CD4+ T cell clones stimulated with biotinylated anti-IgG1 or anti-CD161 (5 µg/ml) which was crosslinked using anti-biotin antibody in presence of plate-bound anti-CD3 (1 µg/ml) and soluble anti-CD28 (2 µg/ml) stimulation for 4 hours (n=6). E) MFI CD161 on CD4+ cells upon stimulation of CD4+CD161+ TIL with peptide-loaded LLT1 expressing B-LCL in ratio B-LCL:T cell of 1:5 and 1:1 and plate-bound anti-IgG1 or anti-CD161 (10 µg/ml) for 24 hours (n=2). F) Histogram overlays showing CD161 staining in control and *KLRB1* edited CD4+CD161+ TIL from OPSCC patients and HPV16-specific CD4+ T cell clones. Numbers in overlay indicate MFI of CD161.

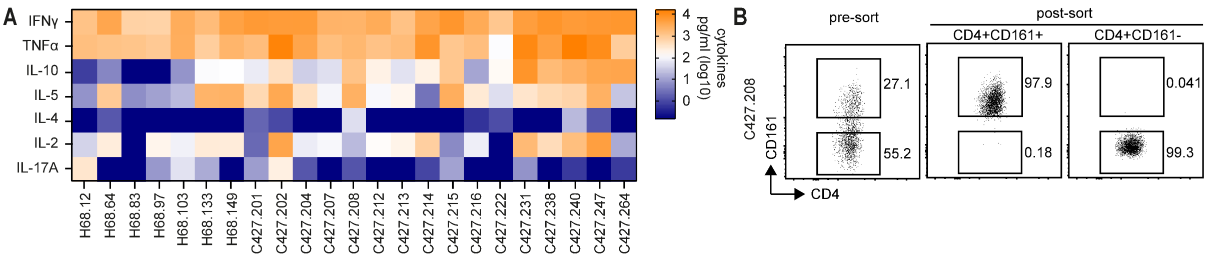

Supplementary Figure 4: Cytokine production by HPV16-specific CD4+ T cell clones and sorting of CD4+CD161+ and CD161- T cell clones
HPV16-specific CD4+ T cell clones from HPV16+IR+ OPSCC (n=7) and cervical cancer patients (n=16) were expanded for 3 weeks. A) Heatmap showing cytokine production (pg/ml, log10) by HPV16-specific T cell clones. B) HPV16-specific CD4+ T cell clones (n=5) belonging to group I (n=2), II (n=1) and III (n=2) were sorted into CD4+CD161+ and CD161- cell populations. Representative dot plots showing sort gating and purity of sorted CD4+CD161+ and CD161- cells.

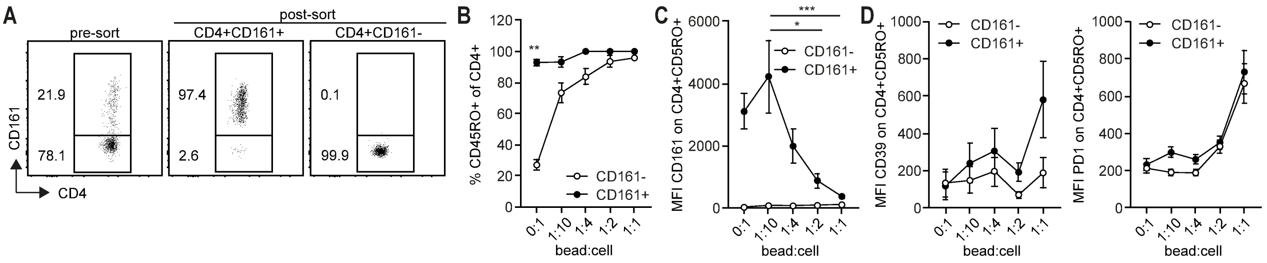

Supplementary Figure 5: TCR signal strength regulates CD161 protein expression

A-D) CD4+CD161+/- cells were sorted from PBMC of adult healthy controls and stimulated for 5 days in presence of anti-CD3CD28 beads as indicated (n=5-9). Summary data represents mean ± SEM. A) Representative dot plots showing gating of CD4+CD161+ and CD161- cells for cell sorting and sort purity. B) Percentage CD45RO+ of CD4+ cells. Significance analyzed for CD161+ compared to CD161- cells. C-D) MFI CD161 (C), CD39 (D) and PD1 (D) of CD4+CD45RO+ cells within CD4+CD161+ (black circles) and CD161- (open circles) cultures. Significance analyzed for bead:cell of 1:10-1:1 compared to 0:1. * p<0.05, ** p<0.01, *** p<0.001.

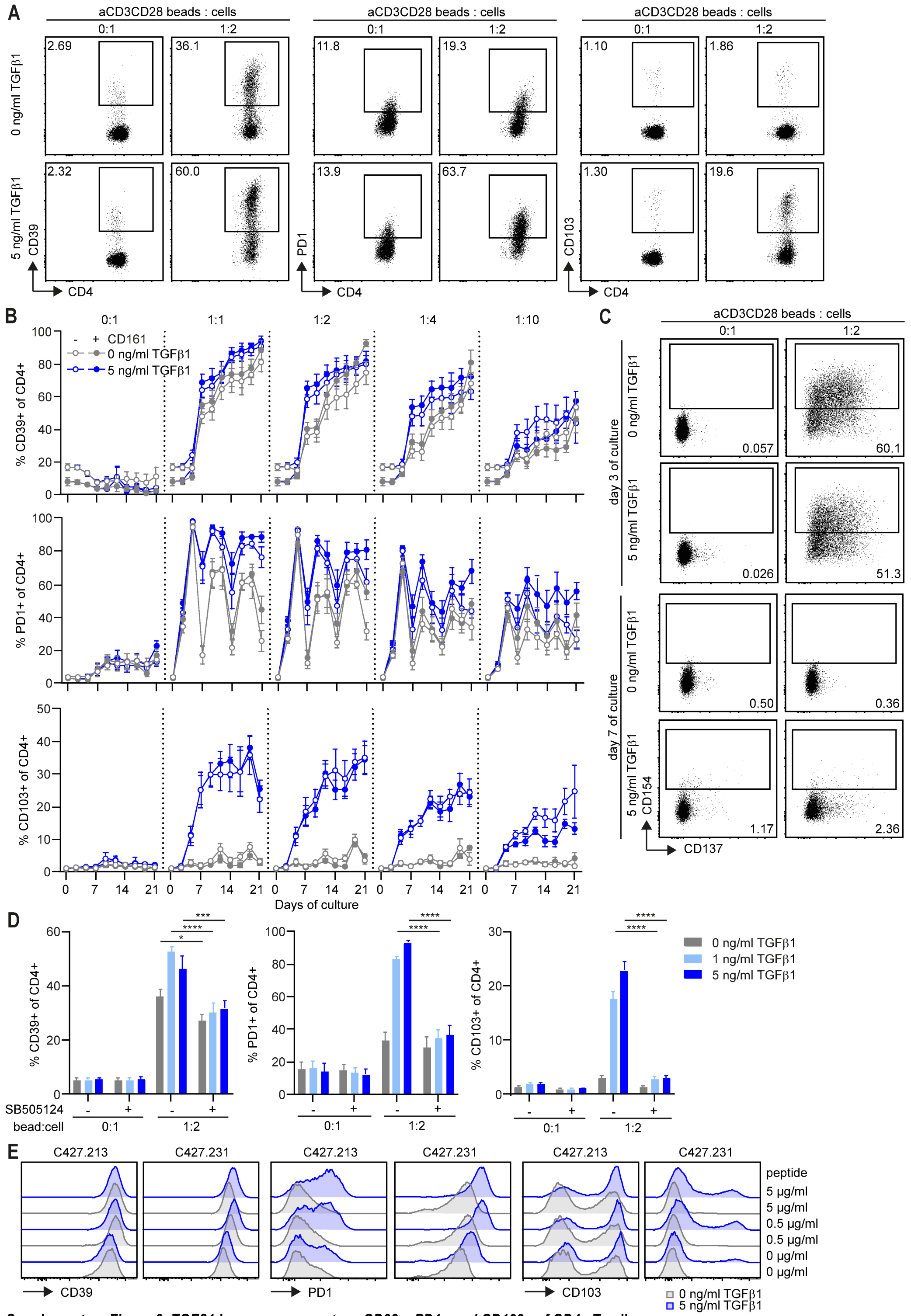

Supplementary Figure 6: TGFβ1 increases percentage CD39+, PD1+ and CD103+ of CD4+ T cells

A-D) CD4+CD45RO+CD161+ and CD161- cells were sorted from PBMC of adult healthy controls and cultured in presence of 0 or 5 ng/ml TGFβ1, 5 ng/ml rhIL-7 and anti-CD3CD28 beads at bead to cell ratios as indicated (n=3-5). Cells were re-stimulated with anti-CD3CD28 every 7 days. TGFβ1 and rhIL-7 were added every 3-4 days. A) Representative dot plots showing percentage CD39+, PD1+ and CD103+ of CD4+ cells within CD4+CD161+ cultures on day 7 of culture. B) Percentage CD39+, PD1+ and CD103+ of CD4+ cells within CD4+CD161+ and CD161- cultures. Lines indicate mean ± SEM. C) Representative dot plots showing percentage CD154+ of CD4+ cells within CD4+CD161+ cultures on day 3 and 7 of culture. D) CD4+CD45RO+CD161+ cells were sorted from PBMC of adult healthy controls, labelled with CTV and cultured in presence of 0,1 or 5 ng/ml TGFβ1, 5 ng/ml rhIL-7, and anti-CD3CD28 beads at bead to cell ratio of 0:1 and 1:2 with or without 1μM SB505124 or DMSO control for 5 days. Summary graphs showing percentage CD39+, PD1+ and CD103+ of CD4+ within CD4+CD161+ cultures (n=5). Lines indicate mean ± SEM. E) Two HPV16-specific CD4+ T cell clones were stimulated with rhIL-15, peptide-loaded B-LCL with or without 5 ng/ml TGFβ1 for 5 days. Histogram plots show CD39, PD1 and CD103 expression on CD4+ cells for indicated stimuli. * $p<0.05$, *** $p<0.001$, **** $p<0.0001$.

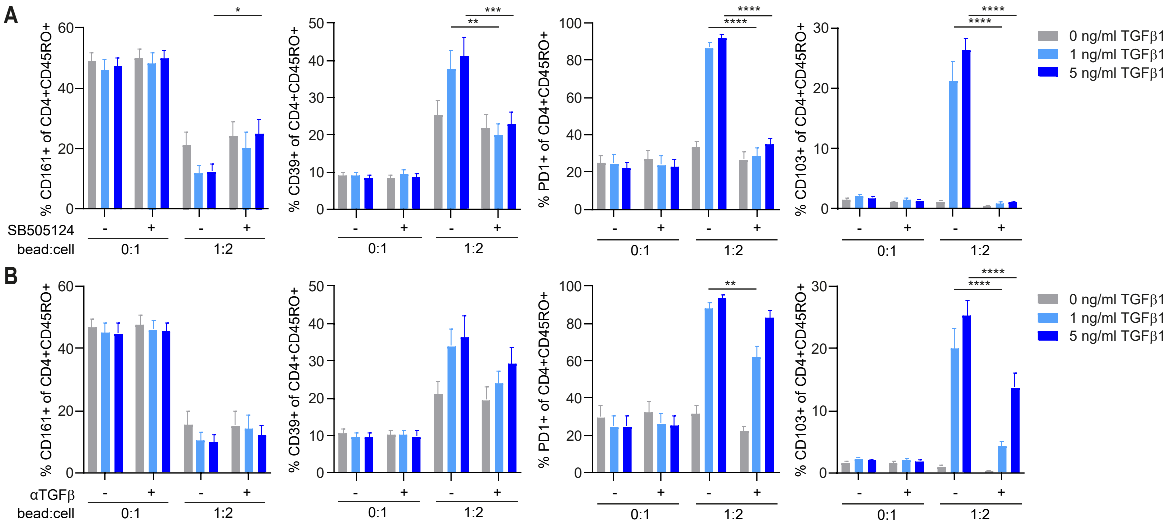

Supplementary Figure 7: Inhibitor of TGFβ receptor signaling and pan-neutralizing TGFβ antibody (partially) reverse the effect of TGFβ1 on CD4+ T cells
A) CD4+ cells from healthy controls were pre-incubated with 1μM SB505124 or DMSO control before stimulation with 0, 1 or 5 ng/ml TGFβ1, 5 ng/ml rhIL-7 and anti-CD3CD28 beads at bead to cell ratio of 0:1 and 1:2 for 5 days (n=4-6). Summary graphs showing percentage CD161+, CD39+, CD103+, PD1+ of CD4+CD45RO+ cells. Graphs show mean ± SEM. B) CD4+ cells from healthy controls were pre-incubated with a pan-neutralizing anti-TGFβ (αTGFβ) or control antibody (10 μg/ml) before stimulation with 0, 1 or 5 ng/ml TGFβ1, 5 ng/ml rhIL-7 and anti-CD3CD28 beads at bead to cell ratio of 0:1 and 1:2 for 5 days (n=4). Summary graphs showing percentage CD161+, CD39+, CD103+, PD1+ of CD4+CD45RO+ cells. Graphs show mean ± SEM. * p<0.05, ** p<0.01, *** p<0.001, **** p<0.0001.

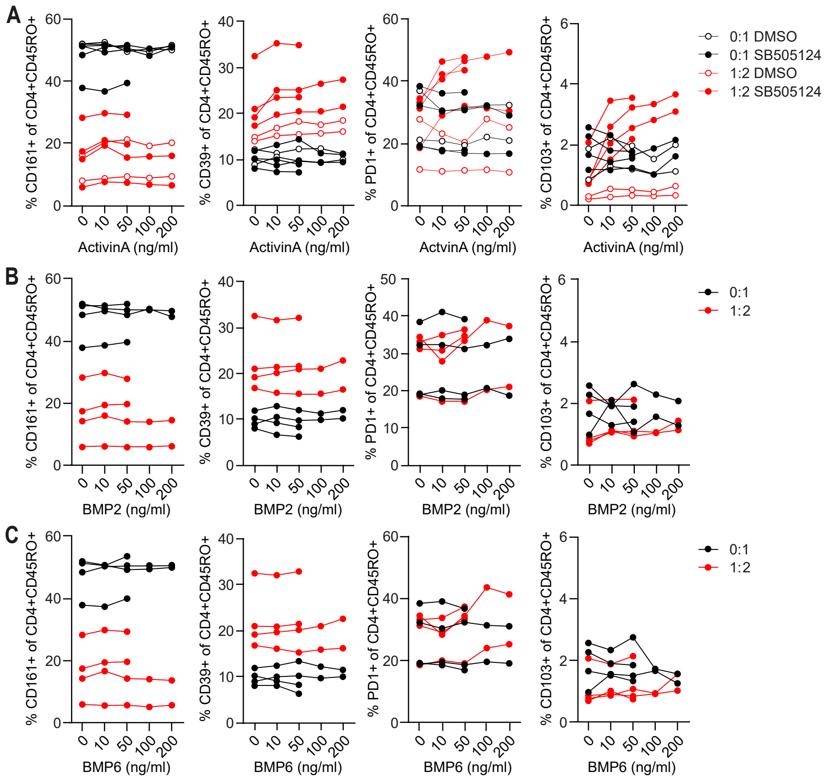

Supplementary Figure 8: Activin A, BMP2 and BMP6 do not affect percentage CD161+ CD4+ cells
CD4+ cells from healthy controls were incubated with 0, 10, 50, 100 or 200 ng/ml Activin A +/- 1µM SB505124 or DMSO control, BMP2 or BMP6, 5 ng/ml rhIL-7 and anti-CD3CD28 beads to cell ratio of 0:1 and 1:2 for 5 days. A) Percentage CD161+, CD39+, CD103+, PD1+ of CD4+CD45RO+ cells upon stimulation with Activin A and SB505124 or DMSO control in presence of anti-CD3CD28 beads as indicated (n=2-4). B-C) Percentage CD161+, CD39+, CD103+, PD1+ of CD4+CD45RO+ cells upon stimulation with BMP2 (B) or BMP6 (C) in presence of anti-CD3CD28 beads as indicated (n=2-4).

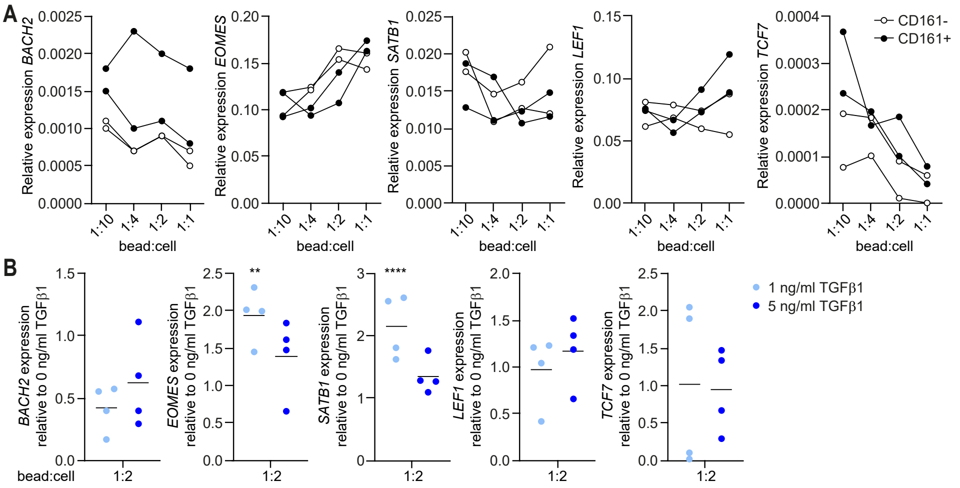

Supplementary Figure 9: Expression of transcription factors associated with T cell differentiation upon TCR and TGFβ1 stimulation
A) CD4+CD161+/- cells were sorted from PBMC of adult healthy controls and stimulated for 5 days in presence of anti-CD3CD28 beads as indicated. Graphs showing relative expression of *BACH2*, *EOMES*, *SATB1*, *LEF1* and *TCF7* within CD4+CD161+ and CD161- cells (n=2). B) CD4+CD45RO+CD161+ cells were sorted from PBMC of adult healthy controls and cultured in presence of 0,1 or 5 ng/ml TGFβ1, 5 ng/ml rhIL-7, and anti-CD3CD28 beads at bead to cell ratio of 1:2 for 5 days. Graphs show *BACH2*, *EOMES*, *SATB1*, *LEF1* and *TCF7* expression in CD4+CD161+ cultures relative to 0 ng/ml TGFβ1 (n=4). Lines indicate mean and significance was analyzed compared to 0 ng/ml TGFβ1. ** p<0.01, **** p<0.0001.

Supplementary Table 1: Antibodies for multispectral immunofluorescence

Marker	Primary antibody clone	Primary antibody dilution	Detection method
CD3	D7A6E	1:50	Alexa Fluor 595 direct
CD8	4B11	1:50	Alexa Fluor 647 indirect
CD161	HPA-039113	1:250	OPAL 520
PD1	D4W2J	1:1000	OPAL 570
CD45RO	UCHL1	1:50	CF 633 indirect
DAPI	-	-	Fluorescent probe

Supplementary Table 2: Flow cytometry antibodies

Marker	Fluorchrome	Clone	Company	Catalog number
CD3	Alexa Fluor 700	UCHT1	BD	557943
CD3	V450	UCHT1	BD	560365
CD3	BV510	UCHT1	BD	563109
CD3	BV510	UCHT1	Biolegend	300448
CD4	AF488	RPA-T4	Biolegend	300519
CD4	APC	RPA-T4	BD	555349
CD4	PE-CF594	RPA-T4	BD	562281
CD8	PE-CF594	RPA-T8	BD	562282
CD8	APC-Cy7	SK1	BD	348813
CD8	BB700	HIT8a	BD	742229
CD39	APC	A1	Biolegend	328209
CD45RA	PerCPCy5.5	HI100	Biolegend	304122
CD45RA	APC-H7	HI100	BD	560674
CD45RO	PE-Cy7	UCHL1	Biolegend	337168
CD103	BV605	Ber-ACT8	Biolegend	350218
CD103	BV650	Ber-ACT8	BD	743653
CD137	APC	4B4-1	BD	550890
CD137	BV421	4B1-1	BD	564091
CD137	BV605	4B4-1	Biolegend	309822
CD154	PE-CF594	TRAP1	BD	563589
CD161	PE	HP-3G10	Biolegend	339904
CCR7	AF488	G043H7	Biolegend	353206
PD1	BV605	EH12.2H7	Biolegend	329924
PD1	PE-Cy7	EH12.2H7	Biolegend	329918
IFNγ	Alexa Fluor 700	B27	BD	557995
TNFα	BV421	MAb11	Biolegend	502932
TNFα	BV605	MAb11	Biolegend	502936
GM-CSF	APC	BVD2-21C11	Biolegend	502310
LLT1	Purified	4F68	Gift VM Braud	n/a
IgG1	Purified	MOPC-21	Biolegend	400102
Goat-anti-mouse IgG	PE	n/a	BD	550589
LIVE/DEAD	Yellow	n/a	Thermo Fisher Scientific	L34959
LIVE/DEAD	Near-IR	n/a	Thermo Fisher Scientific	L34976

Supplementary Table 3: Primers

Gene	Primer sequence 5'-3'	
KLRB1	Forward	AAACAACAGAGAGACCGGGT
	Reverse	TCCAAGGGTTGACAGTGTGA
BACH2	Forward	GGAACGAGCTGCCATGTGAT
	Reverse	GGAGCTATGTGAACGAACGC
EOMES	Forward	GGCGCAAATAACAACAACACC
	Reverse	ATTCAAGTCCTCCACGCCATC
SATB1	Forward	CTCCCCAGGTGAAAACAGCTA
	Reverse	TAACAGCTCGCACAACCATC
LEF1	Forward	GGATCACACCCGTCACACAT
	Reverse	TAGGGTTGCCTGAATCCACC
TCF7	Forward	GCACATGCAGCTATACCCAG
	Reverse	TGAGTTCTCTGGGCCAGTTTG
SOX4	Forward	CCCAGCAAGAAGGCGAGTTA
	Reverse	CATCGGCCAAATTCGTCACC
BACT	Forward	CATCCGCAAAGACCTGTACG
	Reverse	TCTCCTTCTGCATCCTGTCG
B2M	Forward	TGCCGTGTGAACCATGTGAC
	Reverse	CCATGATGCTGCTTACATGTCTCG
SDHA	Forward	CAGGGACCAAAAGAACCGTA
	Reverse	TATCCTGAGCAGCCGAATCT
