# Supplementary material for: CD161 expression and regulation defines rapidly responding effector CD4+ T cells associated with improved survival in HPV16-associated tumors
Source: J Immunother Cancer. 2022 Jan 17;10(1):e003995. doi: 10.1136/jitc-2021-003995 (PMC8765066; doi:10.1136/jitc-2021-003995)
Supplement: Supplementary data [file jitc-2021-003995supp002.pdf]

Supplemental material: Supplementary Methods

CD161 expression and regulation defines rapidly responding effector CD4+ T cells associated with improved survival in HPV16-associated tumors

Chantal L. Duurland[1*], Saskia J. Santegoets[1], Ziena Abdulrahman[1], Nikki M. Loof[1], Gregor Sturm[2], Tom H. Wesselink[3], Ramon Arens[3], Sanne Boekestijn[1], Ilina Ehsan[1], Mariette I.E. van Poelgeest[4], Francesca Finotello[2,5,6], Hubert Hackl[2], Zlatko Trajanoski[2], Peter ten Dijke[7], Veronique M. Braud[8], Marij J.P. Welters[1], Sjoerd H. van der Burg[1*]

[1] Department of Medical Oncology, Oncode Institute, Leiden University Medical Center, Leiden, The Netherlands; [2] Biocenter, Institute of Bioinformatics, Medical University of Innsbruck, Innsbruck, Austria; [3] Department of Immunology, Leiden University Medical Center, Leiden, The Netherlands; [4] Department of Gynecology, Leiden University Medical Center, Leiden, the Netherlands; [5] Institute of Molecular Biology and [6] Digital Science Center (DiSC), University of Innsbruck, Innsbruck, Austria; [7] Department of Cell and Chemical Biology, Oncode Institute, Leiden University Medical Center, Leiden, The Netherlands; [8] Université Côte d'Azur, Centre National de la Recherche Scientifique, Institut de Pharmacologie Moléculaire et Cellulaire, UMR7275, 06560 Valbonne, Sophia Antipolis, France

HPV16-specific CD4+ T cell clones from TIL of HPV16+IR+ OPSCC and cervical cancer patients

HPV16-specific CD4+ T cell clones were previously generated using clonal dilution of tumor infiltrating lymphocytes (TIL) from HPV16+ OPSCC and cervical cancer patients (1, 2). (Sorted) T cell clones were expanded using 10% IMDM (IMDM containing 10% fetal calf serum (FCS, PAA Laboratories), 100 U/ml Penicillin, 100 µg/ml Streptomycin, 2mM L-glutamine, 50µM β-mercaptoethanol) and 10% TCGF, 5 ng/ml rhIL-15, irradiated (7500 RAD) peptide-loaded autologous Epstein Barr Virus (EBV)-transformed B lymphoblastoid cell lines (B-LCL), irradiated (3500 RAD) PBMC from 5 different donors for 3 weeks. Cells were cultured with 10% IMDM, 10% TCGF and 5 ng/ml rhIL-15 once or twice a week as needed. HPV16-specificity and cytokine production was determined using Th1/Th2 cytometric bead array (CBA) kit (BD) and IL-17A ELISA (Thermo Fisher Scientific, TFS) in supernatant obtained from T cell clones restimulated with peptide-loaded B-LCL for 3 days.

Isolation of single-cell tumor digests, bulk culture and expansion of CD4+CD161+/- cells from OPSCC TIL bulk

OPSCC tumor was cut into small pieces and divided into two parts to obtain single-cell suspensions and generate TIL bulk cultures (3). Live CD4+CD8-CD161+/- T cells were sorted from OPSCC TIL bulk cultures and expanded as described for CD4+CD39+/- and CD8+CD103+CD39+/- (4). After expansion, cells were directly used or cryopreserved.

Multispectral immunofluorescence

Multispectral immunofluorescence panel In short, 4µm FFPE tissue sections were deparaffinized, endogenous peroxidase was blocked with hydrogen peroxide and heat induced epitope retrieval was performed with citrate (10mM, pH 6.0). SuperBlock (TFS) was used to block non-specific binding sites. First, antibodies detected by Opal were applied followed by unconjugated antibodies which were incubated overnight. On the second day, after binding of the previous with their corresponding fluorescently labelled secondary antibodies, the directly labeled primary antibodies were incubated for 5 hours, and lastly DAPI staining was applied.

Analysis of multispectral immunofluorescence images Immunofluorescence images were acquired with the Vectra 3.0.5 multispectral imaging microscope (PerkinElmer) at 20x magnification (5). Immune cells in the TME were automatically phenotyped and counted with inForm 2.4 image analysis software (PerkinElmer) after manual training. The software was trained to segment epithelium and stroma, segment DAPI+ nucleated cells, and assign a phenotype to each cell. All phenotypes were visually inspected on accurateness and training was further optimized if errors were detected until all discrepancies were resolved. Given the multitude of possible co-expressed markers, the panel was divided into multiple sub-analyses that contained a small number of markers. The phenotypes of all sub-analyses were merged per cell based on its X,Y-positions enabling the description of the full six marker expression profile for each cell. Immune cell counts were normalized for tissue size (cells/mm^2).

Imaging Mass Cytometry (Hyperion)

Imaging mass cytometry staining Imaging mass cytometry (IMC) was performed using an optimized 33-marker panel (6). FFPE whole tumor sections of 4μm thick were deparaffinized. Heat induced epitope retrieval was performed with citrate (10mM, pH6.0) and SuperBlock (TFS) was used to block nonspecific binding sites. The first mix of 16 heavy metal conjugated antibodies was incubated for 5 hours at room temperature, the second mix of 17 heavy metal conjugated antibodies was incubated overnight at 4°C. Finally, an intercalator (DNA binder) was applied. The tissue slides were then analyzed by IMC using the Hyperion system (Fluidigm). Tumor regions were annotated by a pathologist. The selected regions on the slides were laser ablated and processed through a mass cytometer after which the detected heavy metals and their corresponding antibody were linked to the ablated region with a sub-cellular resolution of 1μm^2 per pixel.

Imaging mass cytometry analysis pipeline The generated high-dimensional output was analyzed by an in-house developed imaging processing pipeline, combining multiple previously validated publicly available software programs (Abdulrahman *et al.* 2021, submitted). The pipeline incorporates 6 automated consecutive steps. Firstly, the signal of the markers was normalized to prevent sample biased clustering as this is frequently encountered when using FFPE tissue. Semi-supervised machine learning was employed to train the software to distinguish signal from background noise for each individual marker using the software programs Ilastik and CellProfiler (7) to crease a binarized mask in which markers with their location on the image were expressed. Secondly, the same semi-supervised machine learning approach was used to segment the tissue into tumor and stroma as well as the individual cells on the images. These masks with layers of required information (normalized signal

BMJ Publishing Group Limited (BMJ) disclaims all liability and responsibility arising from any reliance placed on this supplemental material which has been supplied by the author(s)

for all 33 markers, tissue segmentation, cell segmentation) were then combined in a single FCS file using ImaCyte (in-house developed software program for IMC data analysis, https://github.com/biovault/ImaCytE) (8). HSNE-based cell clustering was performed with Cytosplore (9) and cluster verification and phenotype calling was performed using MCD Viewer and ImaCyte.

Single-cell RNA sequencing and data analysis of OPSCC samples

Live, CD3+CD56+ cells were isolated and dead cells were depleted using a Dead Cell Removal Kit (Miltenyi Biotec) followed by enrichment for CD3+ and CD56+ cells using CD3+ and CD56+ microbeads according to manufacturer's instructions (Miltenyi Biotec). Between 2,108 and 6,107 cells per sample were loaded onto the Chromium Single Cell Controller (10x Genomics), lysed and barcoded. Sequencing libraries were prepared and transcripts were sequenced using HiSeq4000 (Illumina). Sequencing reads were pre-processed and aligned to the GrCh38 reference genome using cell ranger v3.0.0 (10x Genomics). Quality control and downstream analysis was performed using Scanpy v.1.6.0 (10) and Scirpy v.0.6.1 (11). Analysis was restricted to include only CD3+ cells with >700 detected genes, >2000 detected reads, and <11% mitochondrial reads. Cells with >1 TCR-β or >2 TCR-α chains were discarded as putative doublets, in addition to computational doublet detection using Solo (12). In addition, ribosomal, mitochondrial and TCR genes were excluded from downstream analysis. The 6,000 most highly variable genes of 14,242 CD3+ T cells were selected and subjected to unsupervised clustering analysis (13) and visualized using UMAP (14).

References

1. Piersma SJ, Welters MJ, van der Hulst JM, et al. Human papilloma virus specific T cells infiltrating cervical cancer and draining lymph nodes show remarkably frequent use of HLA-DQ and -DP as a restriction element. *Int J Cancer*. 2008;122:486-94. doi:10.1002/ijc.23162
2. Welters MJP, Ma W, Santegoets S, et al. Intratumoral HPV16-Specific T Cells Constitute a Type I-Oriented Tumor Microenvironment to Improve Survival in HPV16-Driven Oropharyngeal Cancer. *Clin Cancer Res*. 2018;24:634-47. doi:10.1158/1078-0432.CCR-17-2140
3. Santegoets SJ, van Ham VJ, Ehsan I, et al. The Anatomical Location Shapes the Immune Infiltrate in Tumors of Same Etiology and Affects Survival. *Clin Cancer Res*. 2019;25:240-52. doi:10.1158/1078-0432.CCR-18-1749
4. Kortekaas KE, Santegoets SJ, Sturm G, et al. CD39 Identifies the CD4(+) Tumor-Specific T-cell Population in Human Cancer. *Cancer Immunol Res*. 2020;8:1311-21. doi:10.1158/2326-6066.CIR-20-0270
5. Abdulrahman Z, de Miranda N, van Esch EMG, et al. Pre-existing inflammatory immune microenvironment predicts the clinical response of vulvar high-grade squamous intraepithelial lesions to therapeutic HPV16 vaccination. *J Immunother Cancer*. 2020;8. doi:10.1136/jitc-2020-000563
6. Ijsselsteijn ME, van der Breggen R, Farina Sarasqueta A, et al. A 40-Marker Panel for High Dimensional Characterization of Cancer Immune Microenvironments by Imaging Mass Cytometry. *Front Immunol*. 2019;10:2534. doi:10.3389/fimmu.2019.02534

7. Schapiro D, Jackson HW, Raghuraman S, et al. histoCAT: analysis of cell phenotypes and interactions in multiplex image cytometry data. *Nat Methods*. 2017;14:873-6. doi:10.1038/nmeth.4391
8. Somarakis A, Van Unen V, Koning F, et al. ImaCytE: Visual Exploration of Cellular Micro-Environments for Imaging Mass Cytometry Data. *IEEE Trans Vis Comput Graph*. 2021;27:98-110. doi:10.1109/TVCG.2019.2931299
9. van Unen V, Hollt T, Pezzotti N, et al. Visual analysis of mass cytometry data by hierarchical stochastic neighbour embedding reveals rare cell types. *Nat Commun*. 2017;8:1740. doi:10.1038/s41467-017-01689-9
10. Wolf FA, Angerer P, Theis FJ. SCANPY: large-scale single-cell gene expression data analysis. *Genome Biol*. 2018;19:15. doi:10.1186/s13059-017-1382-0
11. Sturm G, Szabo T, Fotakis G, et al. Scirpy: a Scanpy extension for analyzing single-cell T-cell receptor-sequencing data. *Bioinformatics*. 2020;36:4817-8
12. Bernstein NJ, Fong NL, Lam I, et al. Solo: Doublet Identification in Single-Cell RNA-Seq via Semi-Supervised Deep Learning. *Cell Syst*. 2020;11:95-101 e5. doi:10.1016/j.cels.2020.05.010
13. Traag VA, Waltman L, Van Eck NJ. From Louvain to Leiden: guaranteeing well-connected communities. *Scientific reports*. 2019;9:1-12
14. McInnes L, Healy J, Melville J. UMAP: Uniform Manifold Approximation and Projection for Dimension Reduction. *arXiv:180203426*. 2018
